# Bracoviruses recruit host integrases for their integration into caterpillar’s genome

**DOI:** 10.1371/journal.pgen.1009751

**Published:** 2021-09-07

**Authors:** Zehua Wang, Xiqian Ye, Yuenan Zhou, Xiaotong Wu, Rongmin Hu, Jiachen Zhu, Ting Chen, Elisabeth Huguet, Min Shi, Jean-Michel Drezen, Jianhua Huang, Xuexin Chen

**Affiliations:** 1 Institute of Insect Science, College of Agriculture and Biotechnology, Zhejiang University, Hangzhou, China; 2 Ministry of Agriculture Key Lab of Molecular Biology of Crop Pathogens and Insects, Zhejiang University, Hangzhou, China; 3 Zhejiang Provincial Key Lab of Biology of Crop Pathogens and Insects, Zhejiang University, Hangzhou, China; 4 UMR CNRS/ Université de Tours 7261 -IRBI: Institut de Recherche sur la Biologie de l’Insecte, Tours, France; 5 State Key Lab of Rice Biology, Zhejiang University, Hangzhou, China; University of Kentucky, UNITED STATES

## Abstract

Some DNA viruses infect host animals usually by integrating their DNAs into the host genome. However, the mechanisms for integration remain largely unknown. Here, we find that *Cotesia vestalis* bracovirus (CvBV), a polydnavirus of the parasitic wasp *C*. *vestalis* (Haliday), integrates its DNA circles into host *Plutella xylostella* (L.) genome by two distinct strategies, conservatively and randomly, through high-throughput sequencing analysis. We confirmed that the conservatively integrating circles contain an essential “8+5” nucleotides motif which is required for integration. Then we find CvBV circles are integrated into the caterpillar’s genome in three temporal patterns, the early, mid and late stage-integration. We further identify that three CvBV-encoded integrases are responsible for some, but not all of the virus circle integrations, indeed they mainly participate in the processes of early stage-integration. Strikingly, we find two *P*. *xylostella* retroviral integrases (PxIN1 and PxIN2) are highly induced upon wasp parasitism, and PxIN1 is crucial for integration of some other early-integrated CvBV circles, such as CvBV_04, CvBV_12 and CvBV_24, while PxIN2 is important for integration of a late-integrated CvBV circle, CvBV_21. Our data uncover a novel mechanism in which CvBV integrates into the infected host genome, not only by utilizing its own integrases, but also by recruiting host enzymes. These findings will strongly deepen our understanding of how bracoviruses regulate and integrate into their hosts.

## Author summary

Parasitoid wasps are a very large group of animals that live in or on the other arthropods. Polydnaviruses (PDVs) are a special kind of DNA virus associated parasitic wasps (primary hosts), which do not replicate in their infected caterpillar hosts (secondary hosts). During parasitoid oviposition, PDVs enter infected hosts and integrate their DNA into the genome of infected hosts, then their virulence genes begin to express and manipulate multiple biological process of hosts to fulfill all the requirements of parasitoids offspring. However, the mechanisms for integration remain largely unknown. Our research found the integration of PDVs is not only by utilizing its own integrases, but also by recruiting host enzymes. Here, we provide a new concept that host intergrases are involved in the integration of PDVs into host genome, and it greatly enhance our understanding of how PDVs regulate and integrate into their hosts, and may also inspire studies on how some DNA viruses may integrate their DNAs into human genome.

## Introduction

DNA viruses use DNAs as their genetic material and generally infect and replicate in host cells. In contrast to the classical DNA viruses, polydnaviruses (PDVs) are a special kind of DNA virus in parasitic insects, which do not replicate in their infected hosts. PDV particles with multiple segments of double-stranded, superhelical DNAs, were firstly observed about 50 years ago [1,2]. They were recognized as a virus family Polydnaviridae in 1991, which was classified into two genera, bracoviruses (BVs) and ichnoviruses (IVs), associated with the two largest parasitoid groups, Braconidae and Ichneumonidae, respectively [3]. PDVs persist as integrated proviruses in the genome of wasp (the primary host of PDVs) germ line and somatic cells [4,5]. PDV assembly and replication occurs only in the nuclei of ovarian calyx cells of female wasps [6]. PDV virions are injected into hosts (the caterpillars of Lepidoptera-moth and butterfly, the second host of PDVs) during parasitoid oviposition. They are reported to infect most of host immune cells, and many other tissue cells as well [7–9], but PDVs do not replicate in host cells [10,11].

PDV genomes consist of two components both residing in the wasp genomes [10]. The first component corresponds to genes of nudiviral origin and codes for proteins involved in particles production [11–13], and the second component is composed of proviral segments used to produce circular dsDNA molecules [14,15]. Only the circular dsDNAs are packaged in viral particles and delivered into caterpillar hosts. PDVs encode virulence genes and microRNAs that can suppress the immune responses and disrupt the development of the parasitized caterpillar hosts thereby enabling wasp development [5,14,16,17]. Thus from a functional point of view, PDVs are gene-delivery vectors, which are used by the wasps to genetically manipulate their host insects [8].

Previous studies showed that BV proviral segments share similar flanking junctions containing the tetramer AGCT, named wasp integration motifs (WIMs) or Direct Repeat Junctions (DRJ), which were identified as the sites where segments circularize following amplification of viral sequences [6,12,13,18–20], thus producing the circular dsDNA molecules packaged in nucleocapsids. It had been suggested that PDV DNAs persist as episomes [21–23] in the caterpillar host, but recent studies revealed also the presence of chromosomally integrated forms in host-derived cultured cells [18,24–26]. WIMs play no roles in the integration of circularized segments into the genome of parasitized caterpillar hosts [18,27]. Another conserved motif corresponding to an inverted repeat domain, named host integration motif (HIM), is found to be involved in the integration processes of some BVs, such as *Microplitis demolitor* Bracovirus (MdBV), *Cotesia congregata* Bracovirus (CcBV) and *Glyptapanteles indiensis* BV (GiBV) [18,25,27,28]. The results from these studies support the hypothesis that the integration of BV circular DNAs into caterpillar host cells take place through a shared HIM-mediated integration process, however, the underlying mechanisms remain largely unknown.

Similarly, except for HIV [29], the integration processes of human viruses are not well known. For example, HHV-6 herpesviruses (HHV) may integrate their DNA into human genome [30,31]. The HHV-6 U94 gene product, which preferentially binds to telomeric DNA sequences and possesses exonuclease/helicase activities, appears to be a good candidate for promoting HHV integration [32] within human telomeres, however cellular factors driving the integration remain unknown [33]. Another example is human papillomavirus (HPV) integration, correlated with cervical cancers, which is supposed to involve host DNA repair mechanism [34].

In this study, we provide a comprehensive analysis of *Cotesia vestalis* bracovirus (CvBV), a polydnavirus of the wasp *C*. *vestalis* (Haliday) that is a larval parasitoid of the diamondback moth, *Plutella xylostella* (Linnaeus), one of the most important pests of cruciferous crops worldwide. We present the expression profiles for each CvBV circle after *C*. *vestalis* infection of the caterpillar host, and find that CvBV circles integrate into the host genome in two distinct ways, i.e., conservatively and randomly. The "conservative-breaking" CvBV circles have host integration motifs (HIMs), and the "random-breaking" CvBV circles have no conservative disruption sites. We show that HIM sequences including intact palindromic regions (8 nucleotides) and border regions (5 nucleotides) are required for conservative integration. We further uncover the underlying mechanisms of CvBV integration into the caterpillar genome by knocking down CvBV-carried integrases and two host enzymes for integration.

## Results

### Updated version of CvBV genome for proviral segments

We updated the current version of CvBV genome for proviral segments, and the new version of the CvBV genome was 505,593 bp and was divided into 30 DNA segments ranging from 3.8 to 38.8 kb (S1 Fig and S1 Table). We identified 218 ORFs including 13 gene families, which are shown in S2 Table. Furthermore, we found that 30 proviral segments resided in 7 scaffolds in the wasp *C*. *vestalis* genome and most CvBV proviral segments (93%) were closely linked to other segments in the *C*. *vestalis* genome (S2A Fig and S3 Table). Boundary sequences of all CvBV segments corresponded exactly to the WIMs, which contained the common tetramer AGCT (S2B Fig).

### Infectious performance of different CvBV circles in their caterpillar host

**Fig 1:**
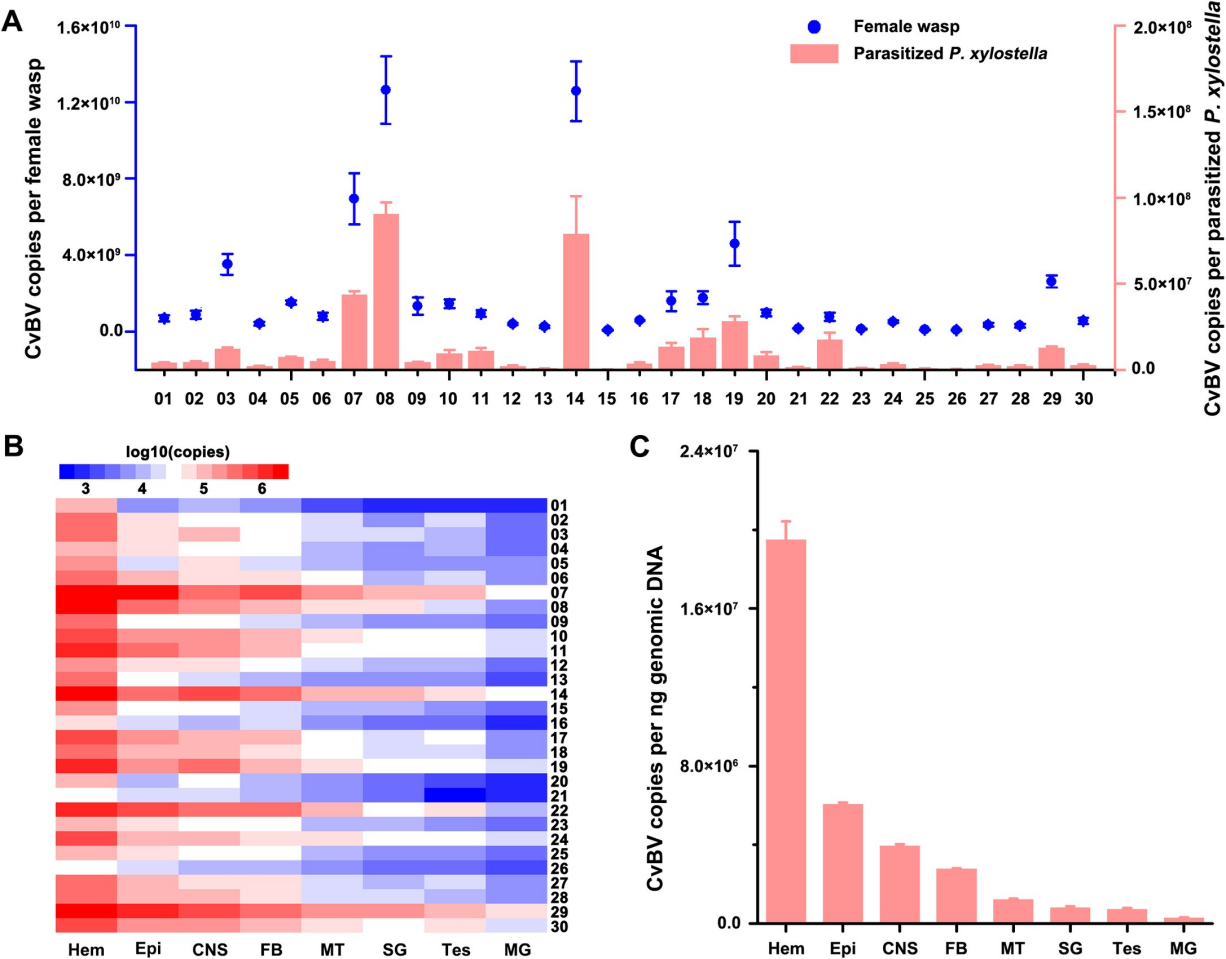
Infection performance of different CvBV circles. **A**: Copies of different CvBV circles in female *C*. *vestalis* wasp (blue dot) and parasitized *P*. *xylostella* host (red columns). **B**: Heat map illustrating the abundance of all 30 CvBV circles in different host tissues. **C**: Copies of different CvBV circles per 1ng genomic DNA isolated from different *P*. *xylostella* tissues at 24 h p.p. Hem: hemocyte, Epi: epidermis, CNS: central never system, FB: fat body, MT: malpighian tubule, SG: silk gland, Tes: testis, MG: midgut. The numbers represent each CvBV circle.

To determine the relationship between CvBV amounts within the wasp ovaries and parasitized host larva, we measured the absolute abundance of all 30 CvBV circles per female wasp and parasitized *P*. *xylostella* at 10 min post parasitization (p.p). As PDV replication occurs only in the nuclei of calyx cells of female wasp ovaries, the number of each CvBV circle per female wasp corresponds to the copies present in the ovaries. Our results showed that total copy number of CvBV reached up to 5.96×10^10^ per female. However, the abundance of CvBV circles varied greatly depending on the circle (Fig 1A) as reported for other PDVs [8,35]. CvBV_08 was the most abundant with 1.26×10^10^ copies accounting for 21.9% of all CvBV circles in the ovaries, and CvBV_15 was the least abundant with 7.42×10^7^ copies, representing 0.15% of the total CvBV circles in the ovaries (Figs 1A and S3B). The relative abundance and ratio of each CvBV circle in the caterpillar host was similar to those observed in wasps (Figs 1A, S3B and S3C) with CvBV_08 being the most abundant, with 9.04×10^7^ copies accounting for 23.07% of the total injected CvBV circles per host, while CvBV_15 was the least abundant, with 2.37×10^5^ copies accounting for only 0.06% (Figs 1A and S3C). Based on their proportion in the female wasps and their hosts, circular CvBV could be classified into 3 groups: high-copy circles (>10% of total circles, i.e. CvBV_07, 08 and 14), median-copy circles (1–10% of total circles, i.e. CvBV_01, 02, 03, 05, 06, 09, 10, 11, 18, 19, 20, 22 and 29) and low-copy circles (<1% of total circles, i.e. CvBV_04, 12, 13, 15, 16, 21, 23, 24, 25, 26, 27, 28 and 30). Each proviral segment is present in single copy in the *C*. *vestalis* genome (S2A Fig) and since we found no obvious relationship between circle abundance and location in wasp genome, our results indicate that non-equimolar segment ratios of circular segments are are mediated by different amplification levels of the corresponding DNAs or selective packaging of the circles.

The WIM region for each CvBV, which is specific, is not used to integrate into the host genome and CvBV do not replicate in *P*. *xylostella*. Thus, the WIM qPCR signal could either represent circular or integrated forms of CvBV. To study the levels of CvBV in different host tissues, we quantified copy numbers for each CvBV in *P*. *xylostella* at 24 h p.p (Fig 1B) using qPCR to amplify the WIM region. The results showed that the amounts for each CvBV in different host tissues also displayed great variations, i.e. CvBV_29 was abundant in all checked tissues. In addition, CvBV_07, 08, 10, 11, 14, 17, 18, 19, 22, 24, 27, 28 and 30 were abundant in epidermis, central nervous system (CNS) and fat body; CvBV_07, 08, 10, 14, 19, 22, 24 and 30 were abundant in midgut; CvBV_07, 08 and 14 were abundant in salivary gland; CvBV_07, 14, 22 and 30 were abundant in testis (Fig 1B). Strikingly, all the CvBV were abundant in host hemocytes (Fig 1B). In general, CvBV abundance differed between tissues, following a gradient of higher to lower abundance in the order: hemocytes, epidermis, CNS, fat body, malpighian tubules, silk glands, testis and midgut (Fig 1C).

### Two distinct integration strategies of CvBV circles

Genomic DNAs were isolated from hemocytes of parasitized *P*. *xylostella* larvae, which was the most infected tissue, and were deep-sequenced to study the integration pattern of each CvBV circle (S4A Fig). A total of 39, 218 chimeric reads containing both nucleotides of CvBV sequence and *P*. *xylostella* sequence were sorted out from 932.5 million clean reads obtained from 3 independent experiments (S4 Table). Those chimeric reads were used to identify a specific motif involved in CvBV integration. After comparing with *P*. *xylostella* genome, we found similar results in terms of integration efficiency and integration regions in 3 independent experiments. After analyzing the respective percentage of intergenic regions and gene regions of *P*. *xylostella* genome, we showed two CvBV circles, CvBV_28 and CvBV_29, preferred to integrate in the intergenic regions of the host genome, while the other circles preferred to integrate in the gene regions (S4B, S4C and S4D Fig). To obtain a global view of the distribution of insertions throughout the *P*. *xylostella* genome, we analyzed *P*. *xylostella* genomic scaffolds using 100-kb-length windows and counted insertion events (IE) within these windows. The global analysis showed that the insertion sites of CvBV are widespread in *P*. *xylostella* hemocyte DNA but not evenly distributed (S5 Fig). And we did not observe a specific shared motif in the *P*. *xylostella* genome near the different insertion sites for any CvBV circles.

**Fig 2:**
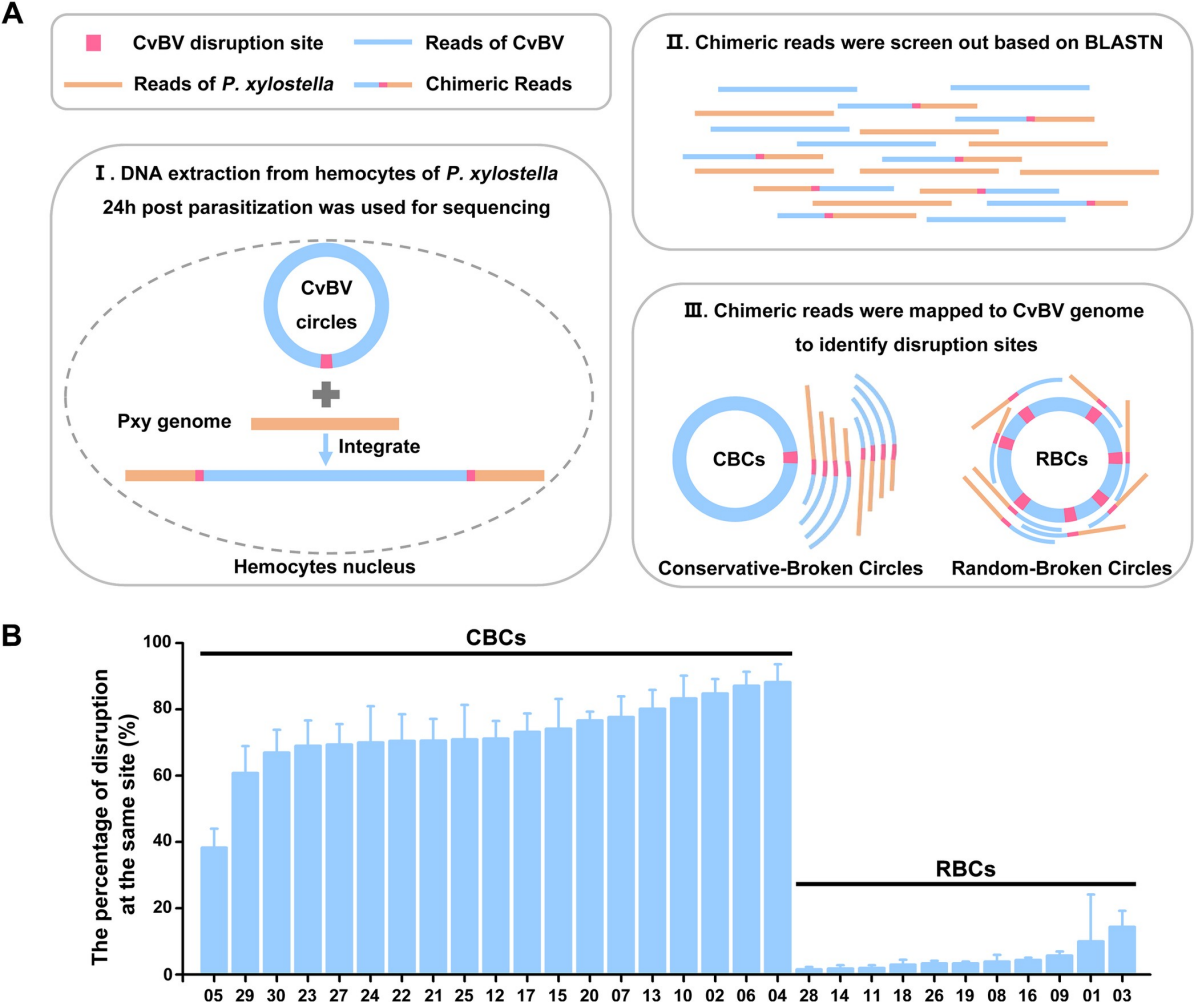
CvBV circles are disrupted in two distinct modes. **A**: Schematic diagram of identifying the disruption sites of CvBV circles. Step I: DNA was extracted from hemocytes of caterpillar hosts 24 h p.p. The DNA used for sequencing consists of CvBV DNA, *P*. *xylostella* DNA and *P*. *xylostella* DNA with integrated CvBV DNA. Step II: Chimeric reads containing both nucleotides of CvBV and *P*. *xylostella* sequences were sorted out based on BLASTN. Step III: Chimeric reads were then mapped to CvBV genome to identify the fractured sites. Two modes of disruption were found for CvBV circles, which included Conservative-Broken Circles (CBCs) and Random-Broken Circles (RBCs). **B**: The ratios of a conserved disruption site for CBCs and RBCs during integration. The abscissa represents different circles of CvBV, and the ordinate represents the percentage of disruption at the same site.

Besides, based on the alignment of chimeric reads on the CvBV genome, we could easily figure out their breaking sites and the resulting patterns. The number of chimeric reads mapping to each CvBV circle was variable (S5 Table), which could be due to the different abundance and integration efficiency of the circles. Interestingly, there are two breaking modes for CvBV integration into the host genome. The first we called "conservative-breaking", which means that the CvBV circles have host integration motifs (HIMs), and the second type we called "random-breaking", which means that the CvBV circles have no conservative disruption sites (Fig 2A). Specifically, 19 CvBV circles (02, 04, 05, 06, 07, 10, 12, 13, 15, 17, 20, 21, 22, 23, 24, 25, 27, 29 and 30) had HIM disruption sites on their circles, which were named as Conservative-Broken Circles (CBCs) (Fig 2B). On the contrary, breaking sites of the remaining 11 CvBV circles (36.67%) were completely random during integration, therefore they were named”Random-Broken Circles” (RBCs) (Fig 2B). Interestingly, CBCs can also integrate randomly into the host genome, whith the following frequencies: 12% of CvBV_04, 13% of CvBV_06, 15% of CvBV_02, 17% of CvBV_10, 20% of CvBV_13, 22% of CvBV_07, 23% of CvBV_20, 26% of CvBV_15, 27% of CvBV_17, 29% of CvBV_12, 29% of CvBV_25, 29% of CvBV_21, 30% of CvBV_22, 30% of CvBV_24, 31% of CvBV_27, 31% of CvBV_23, 33% of CvBV_30, 39% of CvBV_29 and 62% of CvBV_05 (Fig 2B).

### Validation of HIM sequences using a PCR-based detection assay

We randomly selected 3 CBCs, CvBV_02, CvBV_21 and CvBV_22, and used a PCR-based assay to validate their HIMs identified by chimeric reads. The primers were designed to amplify specific segments from each circle (S6A Fig). When using the CvBV DNA as template, PCR products were obtained for all pairs of primers (S6B Fig). In contrast, few or no amplicons, were obtained for CvBV_02-S2, CvBV_21-S6 and CvBV_22-S2 segments using templates isolated from host hemocytes 24 h p.p (S6B Fig). The results suggest that CvBV_02 disrupts into linear DNAs at its S2 region, CvBV_21 at its S6 region and CvBV_22 at its S2 region. during integration. CvBV_02-S2, CvBV_21-S6 and CvBV_22-S2 were further divided into 3 small segments to target the breaking sites, and our results narrowed down the regions within CvBV_02-S2C, CvBV_21-S6B and CvBV_22-S2C segments (S6C Fig). Next, we performed hiTAIL-PCR to identify the precise breaking sites, according to the sequences of CvBV_02-S2C, CvBV_21-S6B and CvBV_22-S2C segments which was found to correspond to the HIM site (S6D Fig). Finally, we found that the HIMs of CvBV_02, 21 and 22 were consistent with those identified by chimeric reads (S1 Data). We also showed that HIMs were different to WIMs, although located closely from 128 to 427 nucleotides (nt) in distance, except for CvBV_05, 07 and 21 (3427 nt, 892 nt and 4600 nt in distance, respectively). The data combined with the results from alignments of chimeric reads and CvBV genome show that CBCs integration is associated with deletion of a stretch of nts (39 to 71 bp) in each CvBV circle (Table 1), and the deleted nts are not conserved (S7 Fig).

**Table 1:** The information of HIM regions for Conservative-Broken CvBV circles (CBCs).

Circle ID	Positions of WIM	Positions of deleted sequences between HIM during integration	Length of deleted sequences (nt)	Proximity length between WIM and HIM (nt)
CvBV_02	1–9	4303–4352	50	220
CvBV_04	1–10	253–291	39	251
CvBV_05	1–12	3982–4031	50	3427
CvBV_06	1–10	7642–7690	49	472
CvBV_07	1–12	9850–9889	40	892
CvBV_10	1–11	12018–12076	59	226
CvBV_12	1–9	12879–12918	40	363
CvBV_13	1–9	180–219	40	178
CvBV_15	1–9	268–307	40	266
CvBV_17	1–9	320–359	40	318
CvBV_20	1–9	17853–17912	60	215
CvBV_21	1–10	4602–4641	40	4600
CvBV_22	1–10	407–445	39	405
CvBV_23	1–10	369–407	39	367
CvBV_24	1–8	172–210	39	170
CvBV_25	1–10	157–206	50	155
CvBV_27	1–10	474–525	52	472
CvBV_29	1–11	133–201	69	131
CvBV_30	1–11	130–201	72	128

### Functional analysis of CvBV HIMs in the integration processes

**Fig 3:**
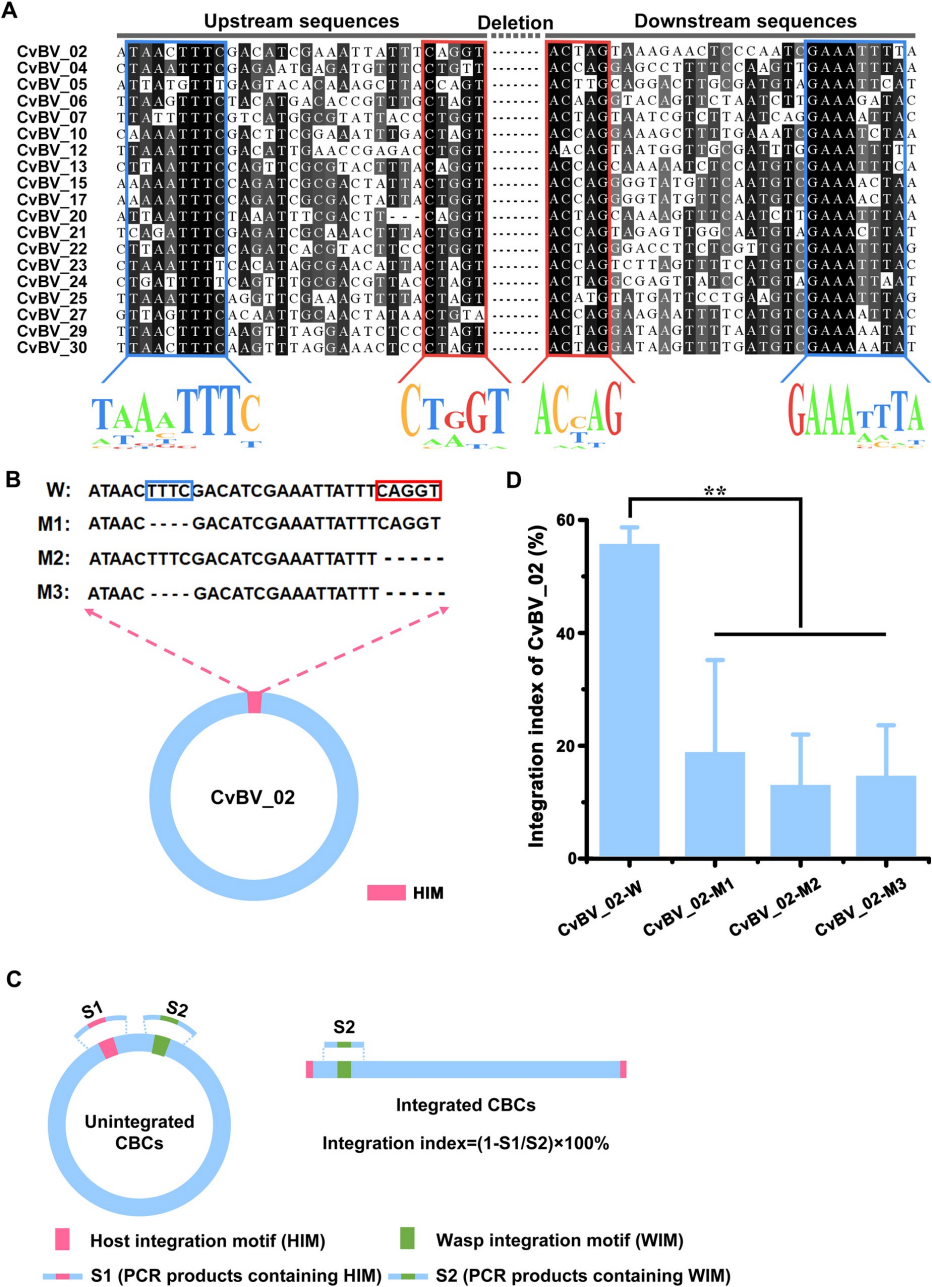
Structural and functional analysis of host integration motifs (HIMs). **A**: The alignment of upstream (left) and downstream (right) sequences around the deletion fragments of the 19 CBCs. The base below represents the most frequently occurring base at this position. Two pairs of boundary sequences forming reverse complementary repeats of 8 nts (TAAATTTC and GAAATTTA) and 5 nts (CTGGT and ACCAG) constituting the borders of the insertions. **B**: Schematic of circle CvBV_02 and its mutants (M1, M2 and M3) in the HIM region. The nucleotide TTTC (in blue square) of wide type (WT) was deleted in M1, nucleotide CAGGT (in red square) of WT was deleted in M2, and both of the sequences were deleted in M3. **C**: Schematic diagram of calculating the integration index of CBCs via amplifying the regions of WIMs and HIMs. The integration index was calculated according to the percentage of reduced amplifying products of HIM regions using two pairs of specific primers across their HIM and WIM regions via qPCR analysis, in which WIM regions were used as internal control. **D**: The integration indices of different CvBV_02 mutants in *P*. *xylostella*. Error bars indicate ± SD. Differences were tested with Tukey-test (**: statistical difference for p < 0.01).

We investigated the structure of CvBV HIMs from all 19 CBCs. Alignment analysis showed that they had two pairs of boundary sequences forming reverse complementary repeats of 8 nts (TAAATTTC and GAAATTTA) and 5 nts (CTGGT and ACCAG) constituting the borders of the insertions (Fig 3A). HIM sequences were similar to those reported in other bracoviruses, such as CcBV and MdBV [18,27]. But the accurate function of HIMs was not experimentally confirmed yet. To determine whether the “8+5” motif is essential for integration, we constructed CvBV_02 circle *in vitro*, which is the smallest circle, and mutated the reverse complementary sites with either deletion of TTTC in the 8 nt region (M1), or deletion of CTGGT in the 5 nt region (M2) or with both deletions (M3) (Fig 3B). The purified recombinant CvBV_02 circular DNA was then injected into host larvae. The integration index of CvBV_02 was calculated according to the percentage of reduced amplifying products of HIM regions using two pairs of specific primers (Fig 3C). We found the integration index of different CvBV_02 mutants in *P*. *xylostella* decreased significantly, when compared with controls (Fig 3D). These results strongly suggest that intact “8+5” HIM sequences are required for CBCs integration processes. Moreover, this shows that HIM mediated integration of CvBV_02 can occur without the presence of virus particles which contain viral integrases.

### CvBV-encoded integrases are involved in the integration of some CBCs

**Fig 4:**
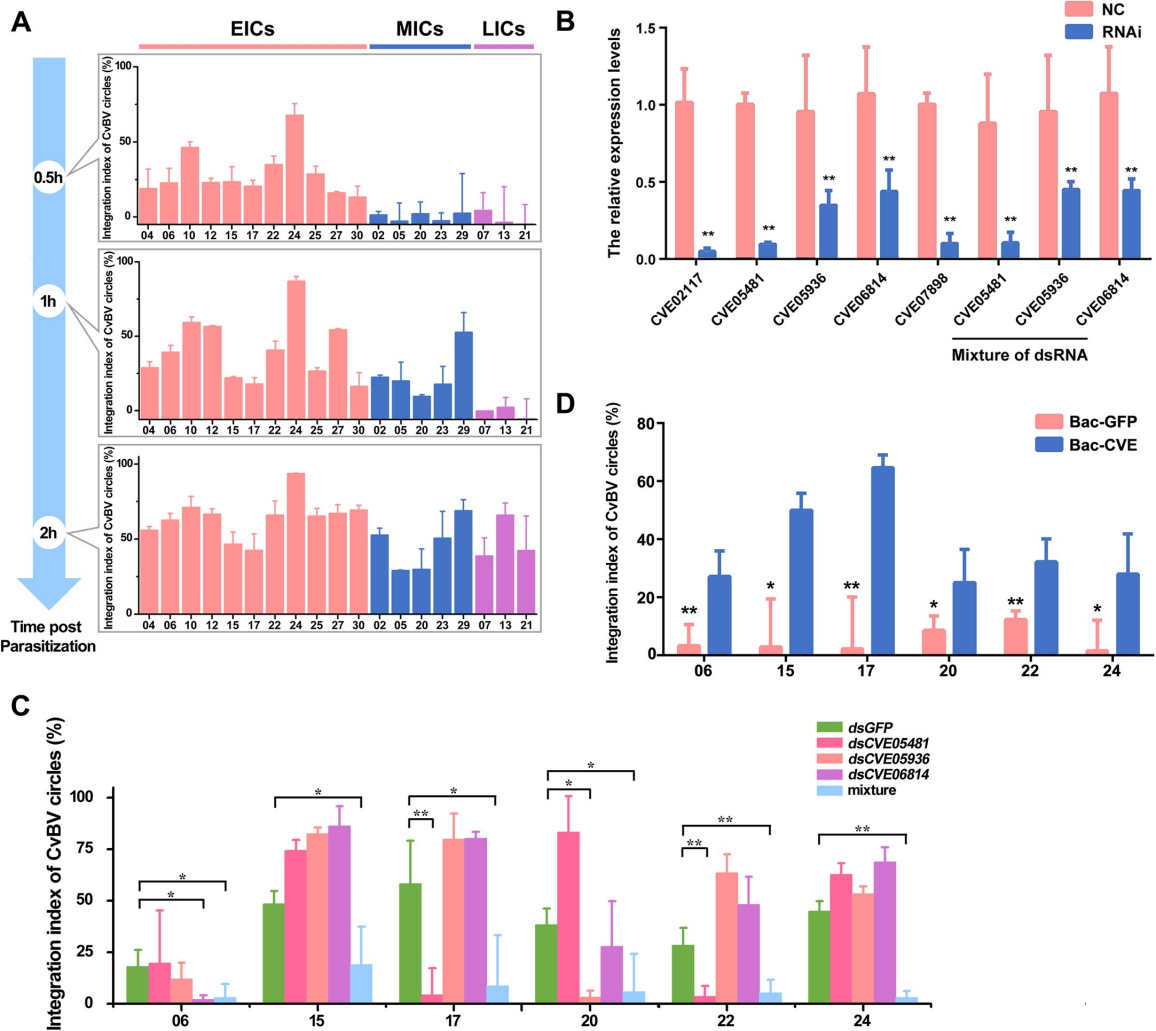
CvBV-encoded integrases are involved in the integration of some CBCs. **A**: Timeline of the integrations of 19 CBCs. The early integrated circles (EICs) integrated within 0.5 h p.p and mid integrated circles (MICs) integrated between 30 minutes and 1 h, and some late integrated circles (LICs) even later (between 1h and 2h). **B**: RNA interference efficiency of CvBV integrase genes (*CVE02117*, *CVE05481*, *CVE05936*, *CVE06814 and CVE07898*). The expression levels of CvBV integrase genes in wasp ovaries were measured by qPCR at the emergence of *C*. *vestalis*. Error bars indicate ± SD. Differences among samples were tested with Tukey-test (**: statistical difference for p < 0.01). **C**: The integration indices of CvBV circles (06, 15, 17, 20, 22 and 24) after parasitism by *C*. *vestalis* whose CvBV integrase genes were interfered. The wasps that were injected with *dsGFP* were used as control. **D**: The integration indices of CvBV circles (06, 15, 17, 20, 22 and 24) after interference of CvBV integrase genes. Bac-CVE means that the CvBV integrase genes were rescued by baculoviruses. The integration indices of CvBV_06, 15, 17, 22, and 24 were detected at 30 min p.p. The decreased integration indices of CvBV_20 were detected at 1 h p.p. Error bars indicate ± SD. Differences among samples were tested with Tukey-test (*: statistical difference for p < 0.05 and **: statistical difference for p < 0.01).

PDVs have been reported to infect the host in early stages of parasitism [21,22], but different PDV circles may not necessarily be integrated at the same time. To assess this hypothesis, we detected the integration index of CBCs at different time points after *C*. *vestalis* infection. According to the time courses of CBCs integrations, we found a progressive increase of CBCs index over time with a maximum reached at 4 h post oviposition (the last time point tested), for all CBC circles indicating that integration occurs within a short time frame after oviposition. However we found that depending on the circle integration can begin very shortly after oviposition within the first 30 minutes, whereas for some circles the rise of the integration slope occurs between 30 minutes and 1 h hour or between 1h and 2h. We thus divided CBC in three categories: early integrated circles (EICs) (CvBV_04, 06, 10, 12, 15, 17, 22, 24, 25, 27 and 30), mid integrated circles (MICs) (CvBV_02, 05, 20, 23 and 29) and late integrated circles (LICs) (CvBV_07, 13 and 21) (Figs 4A and S8).

There is a special group of PDV integrase genes having a nudiviral origin present in the wasp genome. They are known as HzNVORF140 and HzNVORF144 in *Heliothis zea* nudivirus, and the HzNVORF140-like and HzNVORF144 homologues as structural components of MdBV virions are supposed to catalyze PDV integration [5,36]. In this study, we identified three HzNVORF140 and two HzNVORF144 homolog genes (S9 Fig and S2 Data) from the conserved nudivirus-like gene set in proviral CvBV genome. All proviral integrases and all proteins shown in the phylogenetic tree of S9 Fig are related to XerC/D-like site-specific recombinases. One of the HzNVORF144-like proteins (CVE05936) displayed a phage integrase domain (DNA BRE-C) while the other HzNVORF144-like proteins (CVE06814) and 3 HzNVORF140-like proteins (CVE02117, CVE05481 and CVE07898) had no detectable integrase domain, which is similar to what is observed in *C*. *inanitus* and *C*. *congregata* BV [37]. Genomic structure analyses based on 171 insect species (S6 Data) and NCBI’s BLASTP analyses showed that nudivirus integrases are generally conserved in bracovirus-carrying *Cotesia*, *Micropitis* and *Chelonus* wasps. After knocking down each individual gene (Fig 4B), we found that *CVE05481* was involved in CvBV_17 and CvBV_22 integration, *CVE05936* in CvBV_20 integration, and *CVE06814* in CvBV_06 integration (Figs 4C and S10). Inactivation of the three genes also impaired integration of circles already impacted by inactivation of a single gene, as expected. Moreover, the integration ability of CvBV_15 and CvBV_24 was impaired only when the three genes were inactivated, suggesting some redundancy in the three integrase functions (Figs 4C and S10). The integration ability of CvBV_06, 15, 17, 20, 22, and 24 was rescued after the recombinant baculoviruses modified with the insertion of *CVE05481*, *CVE05936*, and *CVE06814* were injected into *P*. *xylostella* before parasitization (Fig 4D). Thus, three CvBV integrase genes *CVE05481*, *CVE05936*, and *CVE06814* play an important role in integration of at least six CBCs. It should be noted that RNA expression was not totally suppressed in particular for CVE05936, and CVE06814, suggesting that obtaining even lower amounts of recombinase might allow to reveal an effect of these recombinases on the integration of other CBC circles.

### Two host integrase genes are essential for integration of 4 CBCs

**Fig 5:**
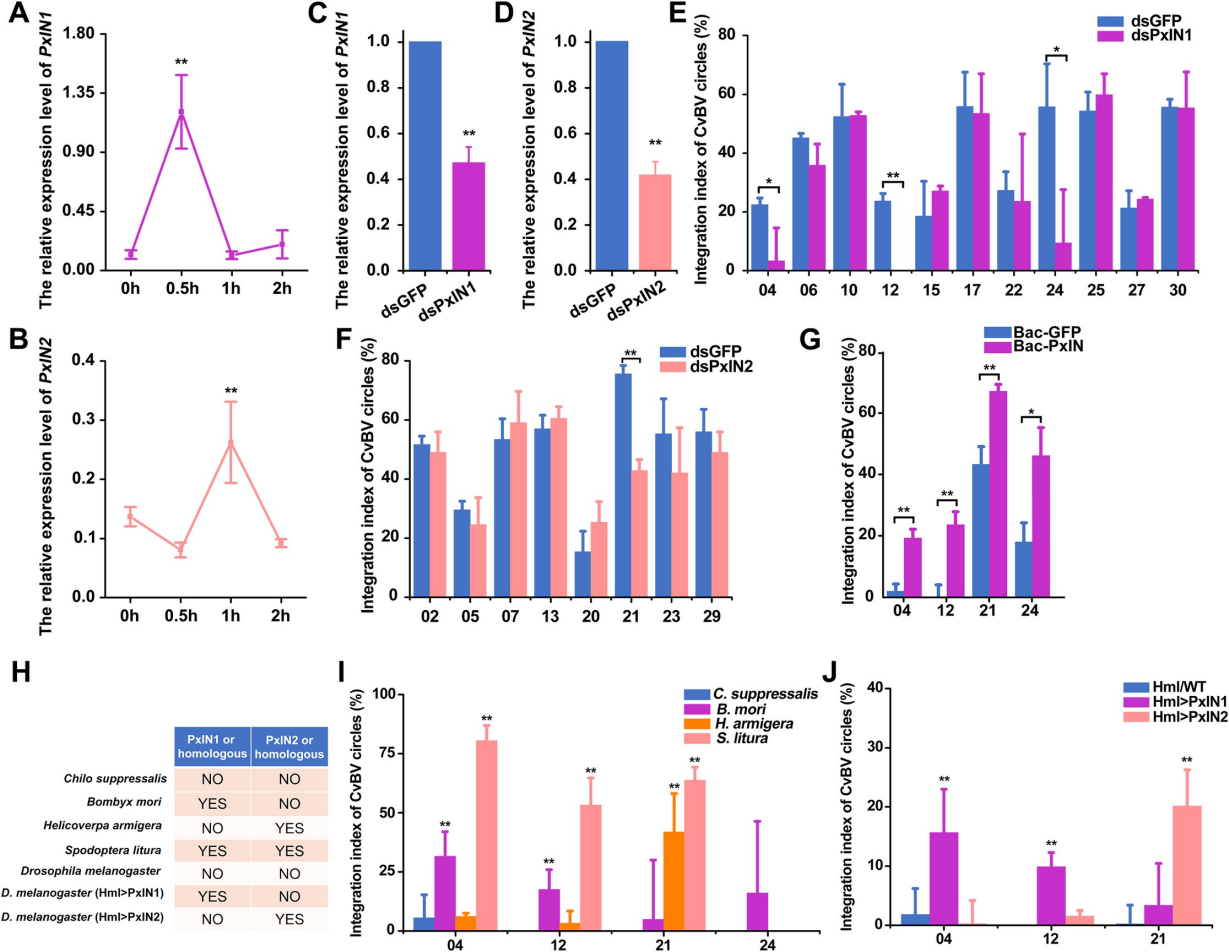
*PxIN1* and *PxIN2* are essential for CvBV integration. **A** and **B**: The transcript levels of host *PxIN1* and *PxIN2* at the early parasitization stage, respectively. **C** and **D**: RNA interference efficiency of host *PxIN1* and *PxIN2*, respectively. **E**: The integration indices of EICs at 30 min p.p after interference of *PxIN1*. **F**: The integration of MICs at 1 h p.p (CvBV_02, 05, 20, 23 and 29) and LICs at 2 h p.p (CvBV_07, 13 and 21) after interference of *PxIN2*. **G**: The integration indices of CvBV_04, 12, 21, and 24 after the interference of *PxIN1* and *PxIN2*. Bac-PxIN means that *PxIN1* and *PxIN2* were rescued by baculoviruses. The integration indices of CvBV_04, 12, and 24 were detected at 30 min p.p. The decreased integration indices of CvBV_21 were detected at 2 h p.p. **H**: The distribution of *PxIN1*, *PxIN2* and their homologues in CvBV non-adaptive hosts and ectopic expression *PxIN1* and *PxIN2* in *D*. *melanogaster*. **I** and **J**: Detection of the integration indices of CvBV_04, 12, 21 and 24 into genomes of non-adaptive caterpillar hosts (i), and flies that ectopically expressed *PxIN1* and *PxIN2* (j). One-way ANOVA followed by Tukey’s multiple comparison test for D, G, E, H and I. Error bars indicate ± SD, **p<0.01, *p<0.05.

In order to assess whether host factors might also be recruited following viral infection to contribute to CBCs integration, we analyzed the transcriptomes of the *P*. *xylostella* hemocytes versus *C*. *vestalis* infected host hemocytes, and found 1747 genes were upregulated and 1629 genes were downregulated (S11A Fig). Gene ontology enrichment analysis showed that there were 22 genes associated with DNA integration (15 up, 7 down), and KEGG enrichment analysis revealed 45 genes involved in DNA repair (9 up, 36 down) (S6 Table, S3 and S4 Data). Furthermore, we analyzed the transcriptional dynamics of the 15 up-regulated genes associated with DNA integration in *P*. *xylostella* (S11B Fig). Interestingly, we observed that transcriptional upregulation of two genes was timely correlated with the integration kinetics of CBCs, respectively: one gene increased rapidly at 30 min p.p (Fig 5A) and the other increased significantly at 1 h p.p (Fig 5B). According to sequence alignments and gene functional annotation, the genes contained 2 conserved domains (integrase zinc-binding domain and integrase catalytic core domain) of integrases. Thus, they refered to retroviral integrases and were named *P*. *xylostella integrase 1* (*PxIN1*) and *P*. *xylostella integrase 2* (*PxIN2*), respectively. The expression pattern of *PxIN1* was related to the EICs, while the transcript pattern of *PxIN2* was related to the MICs and LICs. After *PxIN1* interference (Fig 5C), we found the integration of 3 EICs (CvBV_04, 12 and 24) were significantly suppressed at 30 min p.p (Fig 5E). In the case of interference of *PxIN2* (Fig 5D), the integration of LIC, CvBV_21, was significantly suppressed (Fig 5F). But the integration of CvBV_04, 12, 21, and 24 were rescued after the recombinant baculoviruses modified with the insertion of *PxIN1* and *PxIN2* to resistant to RNAi were injected into *P*. *xylostella* before parasitization (Fig 5G). These results indicate that PxIN1 is involved in CvBV_04, 12 and 24 integration, while PxIN2 is involved in CvBV_21 integration, which could reflect a more general role in EIC and LIC integration respectively provided complete integrases suppression could be reached.

### Heterologous infection in non-host insects further suggests the implication of PxIN1 and PxIN2 integrases in CBCs integration

Genomic survey in insects indicated that PxIN1 and PxIN2 are present in other Lepidoptera (S11C Fig). Similar to *P*. *xylostella*, both PxIN1 and PxIN2 were found in *Spodoptera litura*, while only PxIN1 was found in *Bombyx mori* and only PxIN2 was found in *Helicoverpa armigera*. Contrastingly, the genes were not detected in the genomes of *Chilo suppressalis*, or of the Diptera *Drosophila melanogaster* (Fig 5H). *S*. *litura*, *B*. *mori*, *H*. *armigera*, *and C*. *suppressalis*, not naturally parasitized by *C*. *vestalis*, were used to further assess the roles of PxIN1 and PxIN2 in CvBV integration by artificial injection of CvBV particles. CvBV_04, 12 and 21 were found to be integrated into *S*. *litura* DNA at 24 h post injection (p.i) of 0.05 FE CvBV, while no circles were integrated into DNA of *C*. *suppressalis* (Fig 5I), lacking PxIN1 and PxIN2 homologues. Furthermore, we also found that CvBV_04 and CvBV_12 showed integration into the DNA of *B*. *mori* and CvBV_21 was integrated into DNA of *H*. *armigera*, respectively (Fig 5I). However, whereas we found that PxIN1 was involved in CvBV_24 integration in *Plutella xyllostella*, we did not find CvBV_24 integration into the DNA of *B*. *mori* and *S*. *litura* (Fig 5I). Next, we used *Hml-GAL4*, a hemocyte specific expression driver, to drive *UAS-PxIN1* and *UAS-PxIN2* in *D*. *melanogaster* hemocytes. 24 h after CvBV virions were injected into fly larvae, CvBV_04 and CvBV_12 were found to be integrated into ectopic *PxIN1* expression fly hemocytes and CvBV_21 was found to be integrated into ectopic *PxIN2* expression fly hemocytes (Fig 5J).

## Discussion

PDVs persist as integrated proviruses in the genome of parasitoid wasps [4,5,10]. The proviral segments of BVs are delineated by short, direct repeat junctions containing the tetramer AGCT, which are called WIMs or DRJs. The WIMs were identified as the sites where segments circularize during replication [12,13,18,19]. Here, we show that CvBV genome is divided into 30 DNA segments located in the *C*. *vestalis* genome by WIMs containing the tetramer AGCT, which is in accordance with other wasp BVs. It is not surprising that all BV proviral segments share a common circularization mechanism because they originate from a common nudivirus ancestor [5,38] which like related baculoviruses may have to resolve concatemers of genomes produced during replication using a recombinase [39].

It is important to measure the abundance of PDV circles because unlike genomes of pathogenic viruses they do not replicate within caterpillar host. Moreover, circles abundance indirectly reflects the level of expression of virulence genes during parasitism, the most highly expressed genes being encoded by the more abundant circles, although these circles might also contain genes expressed at a low level [35]. We determined the copy numbers of all CvBV circles in wasp ovaries and in the parasitized host by absolute qPCR technique. There were huge variations in the abundance of 30 CvBV circles in the ovaries as reported for MdBV and CcBV [8,35]. For example, three highly produced CvBV circles (CvBV_07, 08 and 14) accounted for more than 50% of the viral DNAs in wasp ovaries. We also compared the copy number of CvBV circles per female wasp with that in parasitized host, and found that parasitism did not modify the relative abundance of CvBV circles produced by females. The production of BV virions begins with amplification of the proviral genome in calyx cells, which is followed by the de novo assembly and packaging of virions [14]. Each proviral segment exists as a single copy in *C*. *vestalis* genome, thus the different amounts of circles depend on the amplication or packaging of the circles, which varies depending on the location of the corresponding proviral segments in the wasp genome, which results in nonequimolar circles in the ovaries [40,41]. This strategy may allow to adjust gene dosage [23,42]. Indeed, higher amounts of viral genes can be delivered into hosts by the more abundant CvBV circles [8,35,43].

PDVs have been reported to persist as chromosomally integrated forms in host-derived cultured cells after infection [18,24–26]. Two MdBV circles and eight CcBV circles have been formally demonstrated to be integrated in host genomic DNA after natural parasitization [18,27]. Integrations of these BV circles involve a host integration motif (HIM) [18,27]. In this study, we sequenced the genomic DNA of parasitized *P*. *xylostella* larval hemocytes to study CvBV integration sites and the mechanism involved in integration. Integration events of CvBV circles were widely spread in host DNA and some showed a preference for gene regions, which is similar to what was observed for CcBV circle integration in *M*. *sexta* hemocyte DNA [27]. Moreover, we found 19 CvBV circles conservatively disrupted at the HIM region during integration, that we named CBCs. Almost all protein tyrosine phosphatase (PTP) family genes (31 out of 33) and viral ankyrin (VANK) family genes (all 5) were localized within these 19 CBCs, which is consistent with results found in CcBV integrated circles [34], but in contrast to MdBV circles that all have a HIM motif DNA [18], suggesting some circles have lost HIM sites in the *Cotesia* lineage. The structure of HIMs (“8+5” oligo-nucleotides motif) contained two reverse complementary sites separated by a non conserved region of approximately 50 nucleotides as described previously [18,27], however its function was not experimentally confirmed yet. In this study, each reverse complementary site was indispensable for integration since mutations in either or both sites limited the integration effciency of CvBV-2 circle. HIM and WIM of the same circle were generally located in a close proximity with one another, which is also consistent with the results in the studies of CcBV and MdBV [18,27]. Moreover, we found the remaining 11 CvBV circles had no HIMs and were broken randomly during integration, that we named RBCs. The number of chimeric reads from 19 CBCs are 32, 665 (83.3%) and the number of chimeric reads from 11 RBCs are 6, 553 (16.7%) (S5 Table), which suggest that most integrated CvBV circles are CBCs.

Genes involved in PDV circles integration have not been identified, though some PDV integrase genes inherited from the nudivirus ancestor are supposed to be involved in the process [5,35]. Some genes belong to the familly of XerC/D tyrosine recombinases of bacteria, the binding site of which share a palindromic structure similar to the HIM site, made of inverted repeats separated by a spacer. Such recombinases form multimeres during their interaction with DNA [44]. By reducing the level of the mRNAs from 3 out of 5 candidate genes using RNAi we found the integration of 4 CBCs was impaired, showing that corresponding gene products were involved in their integration. The HIM sequences of CBCs are similar, but not all 19 CBC integrations were impaired. The adjacent sequences of HIMs, which are different, may play a role in viral integration. Moreover, the treatment down regulating simultaneously the expression of the three genes confirmed the effect on the 4 CBC circles and impacted the integration of 2 additional CBCs, suggesting a redundancy in the function of different integrases.

**Fig 6:**
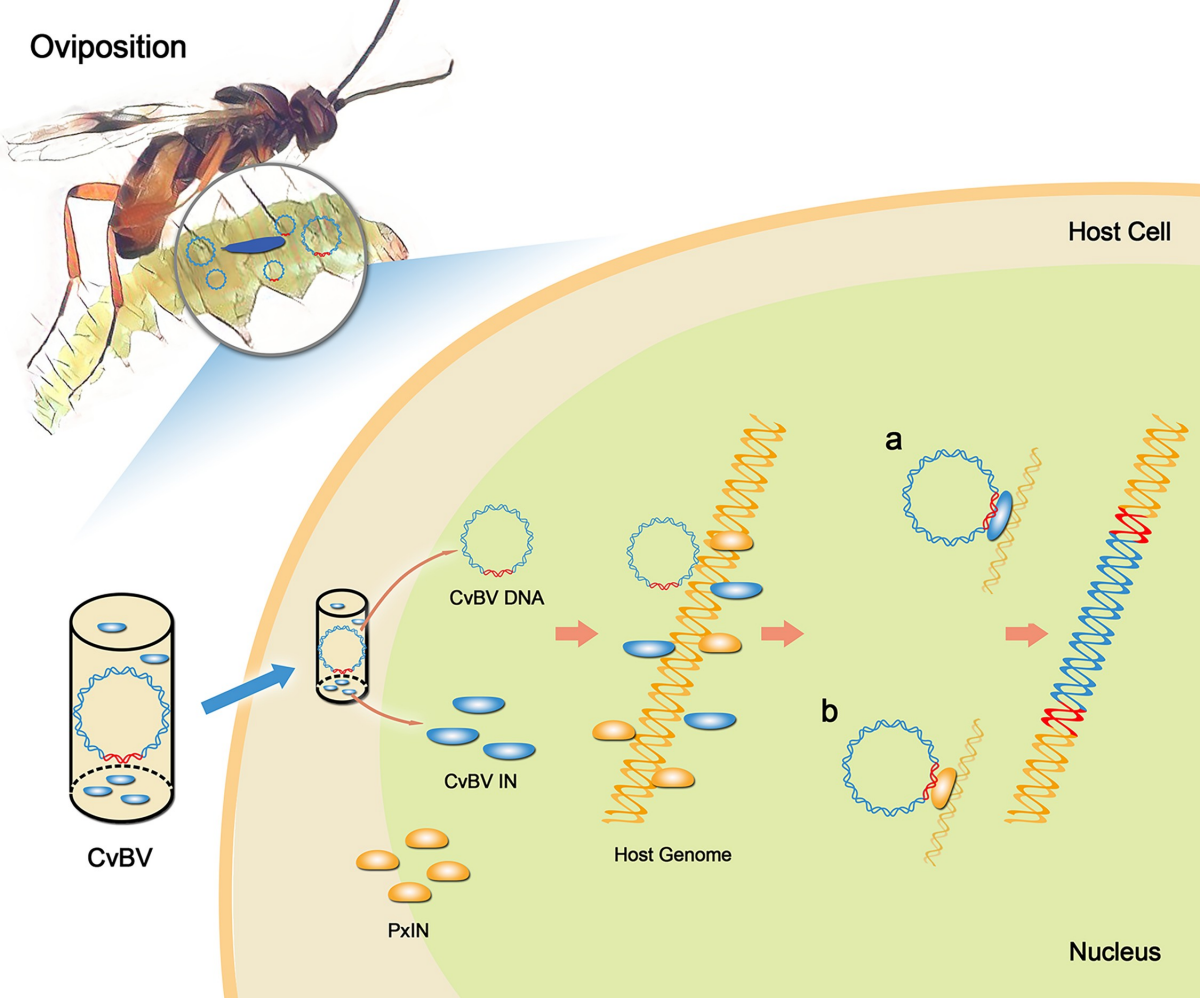
Model for *C*. *vestalis* bracovirus integration into caterpillar genome. Schematic diagram of a model for the processes of how the bracovirus DNA circles integrate into host genome via recruitment of viral or host genes. *Cotesia vestalis* bracovirus (CvBV) are injected into host along with the egg during oviposition. CvBV circles with HIM integrate conservatively into caterpillar genome in two ways. Type a, viral integrase-dependent: Three CvBV-encoded integrases (CvBV INs) CVE05481, CVE05936 and CVE06814 are responsible for the integration of CvBV_06, 15, 17, 20 and 22. Type b, host integrase-dependent: two host integrases (PxIN), PxIN1 and PxIN2 are induced over time and responsible for the integration of CvBV_04, 12 and 21.

Considering that a significant amount of mRNA remains after treatment which can lead to different integrase proteins incorporation into virus particles we hypothesize that the non significant effect of RNAi on 13 other CBCs does not exclude that these genes are also involved in their integration. Strikingly, we also found that two lepidopteran host integrase genes PxIN1 and PxIN2 were involved in the integration of some other CBCs, such as CvBV_04, 12, 21 and 24. PDVs can infect many insect cell lines [45], and we found that the homologues of PxIN1 and PxIN2 in other Lepidoptera caterpillars could help CvBV_04, 12 and 21 to integrate into non-host genomes, and the function of PxIN1 and PxIN2 was also confirmed in the ectopic-expression transgenic flies. Taken together, we propose a model for the mechanisms of how the *C*. *vestalis* bracovirus might integrate into the host genome (Fig 6). Most CBCs with HIM integrate into the caterpillar genome in two ways. Type a, viral integrase-dependent: CvBV-encoded integrase CVE05481, CVE05936, and CVE06814 are injected into host upon wasp oviposition, as components of bracovirus particles and they are necessary to trigger the integration of some early integrated CBCs; Type b, host integrase-dependent: host integrases PxIN1 and PxIN2 are induced quickly after oviposition and sequentially trigger the integration of some other early integrated CBCs and one late integrated CBC circle. In Type a, the CvBV-encoded integrases, as a kind of phage integrases, may complete integrations of some CBCs via a two-step reaction catalyzed by a complex containing two or four monomers of integrases bound to two recombination sites, which is similar to XerC/D recombination model [46]. In Type b, the DNA cutting and strand transfer reactions catalyzed by the retroviral integrases may proceed through phosphodiester transesterification without formation of covalent protein−DNA intermediates, which is similar to retroviral DNA integration [47]. The integration of the remaining CBCs may be associated with some other integrase-like proteins, as there are other homologues of PxIN1 and PxIN2 in *P*. *xylostella* genome (S11D Fig) whereas the integration of RBCs, which account for a small percentage of integrated CvBV circles, would be associated with DNA repair machinery. PxIN1 and PxIN2 were derived from retroviruses (S12 Fig), which may be the reason that host integrases are involved in viral integration. However, those constitutively expressed integrases in *P*. *xylostalla* may be also involved in integration, and it is necessary to be investigated in our further studies. The model we propose directly report the experimental data we obtained and might appear rather complex for the integration of CBC circles sharing a conserved motif. It should be noted that CVE05481 and CVE06814 carrying no integrase domain play a role in CBC integration. In the CvBV integration processes, these two proteins may need a partner containing integrase domain. However it will probably evolve into a more unified one providing more complete knockout of integrase genes can be achieved, for example using Crisper/Casp9 mediated gene deletions. We hope this study will stimulate experiments toward this aim deepening the understanding of CvBV integration processes and the role of host co-factors.

In summary, our results provide the first experimental insights into the molecular mechanisms by which PDVs integrate into host genomic DNA. The remarkable findings that PDVs recruit host enzymes for their integration will deepen our understanding of how parasites regulate their hosts for integration. More broadly, it may also inspire studies on how some DNA viruses may integrate their DNAs into human genome, with potential medical applications against viruses and virally-induced cancers.

## Materials and methods

### Insect rearing

*P*. *xylostella* and its endoparasitoid *C*. *vestalis* were reared as previously described [15]. They were maintained at 25 ± 1°C, 65% relative humidity, and 14 h light: 10 h dark. Adult wasps were fed with 20% honey/water (V/V). Middle 3^rd^ instar *P*. *xylostella* host larvae were individually exposed to a single *C*. *vestalis* female within a 10 mm **×** 80 mm tube to ensure 100% parasitization.

### CvBV genome resequencing and annotation

1000 2-day-old female wasps were dissected and the PDV virions and viral DNA were collected as previously described [15]. Long read single molecule real time sequencing (SMRT) strategy was used to sequence CvBV genome on PacBio Sequel platform (Pacific Biosciences, Menlo Park, CA, USA) with P6 polymerase binding and C4 chemistry kits. For PacBio sequencing, we constructed library of 10 kb using the standard protocol. The long read SMRT sequencing data were corrected using CANU (http://canu.readthedocs.org/) with default parameters. After that, the subreads were used to assemble by SOAPdenovo2, and the result was polished using Quiver (http://www.pacbiodevnet.com/Quiver/).

Putative open reading frames (ORFs) were predicted using FGENESH (http://linux1.softberry.com/berry.phtml) and GENSCAN (http://genes.mit.edu/GENSCAN.html). Database searches were performed using NCBI’s BLASTN, BLASTX, and BLASTP (www.ncbi.nlm.nih.gov/BLAST/). Functional annotation of ORFs was performed through searching against GenBank’s non-redundant (nr) protein database using BLASTP.

### Absolute quantification of CvBV circles

WIMs (wasp integration motifs) of 30 CvBV proviral segments were obtained by mapping CvBV genome to *C*. *vestalis* genome (GenBank No. LQNH00000000). We validated 30 WIMs by PCR using specific primers (S5 Data). Quantitative PCR (qPCR) was used to determine the amounts of different CvBV circles in wasp ovaries and parasitized host larvae. An absolute standard curve was generated via PCR amplification of 30 WIMs using specific primers (S5 Data), and these primers that flanked the WIMs on segments were diagrammatized in S3A Fig Standard curves were generated followed by determination of copy numbers from serially diluted amounts of each plasmid standard. qPCR assay was conducted as previously described [48]. The qPCR reactions were conducted on a CFX Connect real time system (BIO-RAD, Hercules, CA, USA) using THUNDERBIRD qPCR Mix (Toyobo, Osaka, Japan). Each qPCR reaction was performed for at least three biological replicates under the following conditions: 95°C for 60 sec, 40 cycles of 95°C for 15 sec and 60°C for 30 sec. Melting curve analyses were performed to ensure that amplified products were specific.

Different tissues of *P*. *xylostella* 24 h post parasitization (p.p) were dissected in PBS (pH 7.2). Epidermis, silk gland, fat body, midgut, hemocytes, central nervous system (CNS, brain and ventral nerve cord), malpighian tubule (MT) and testis were collected, respectively. Hemocytes were collected by bleeding larvae 24 h p.p from a cutting proleg. Genomic DNAs from 2-day-old female wasp, *P*. *xylostella* (10 min p.p) and different tissues from *P*. *xylostella* (24 h p.p) were isolated using the Puregene Core kit (Qiagen, Hilden, Germany). The standard curves (S7 Table) were used to calculate the copy number of CvBV circles.

### Genome resequencing of parasitized *P*. *xylostella* hemocytes and data analysis

Hemocytes of about 500 *P*. *xylostella* larvae (24 h p.p) were collected as one group. Genomic DNA from 3 independent groups was isolated using the Puregene Core kit (Qiagen). DNA concentration was detected by NanoDrop spectrophotometers (Thermo Fihser, MA, USA). A total amount of 1μg genomic DNA per group was used as input for the library preparation. The sequencing libraries were generated using the VAHTS Universal DNA Library Prep Kit for Illumina (Vazyme, Nanjing, China) following manufacturer’s recommendations and were sequenced on an Illumina Hiseq X Ten platform with 150bp paired-end module.

A total of 932.5 million clean reads were obtained from the 3 Illumina runs (S4 Table). Chimeric reads containing both nucleotides of CvBV sequence and *P*. *xylostella* sequence were screen out based on BLASTN (E-value <10^−5^) against CvBV genome and *P*. *xylostella* genome [49]. In a stringent analysis, only reads aligned with at least 28 nucleotides were kept to avoid incorrect mapping due to short alignments. A total of 39, 218 chimeric reads referred to 30 CvBV circles were obtained (S4 Table). Reads were then mapped again to CvBV genome to identify disruption sites of CvBV circles, and to *P*. *xylostella* genome to identify integration sites.

When the integration sites of each CvBV circle were identified, the percentage of different integration locations (at *P*. *xylostella* gene region or intergenic region) of each circle was calculated according to the counts among the total chimeric reads referred to each CvBV circle. When the disruption sites of each CvBV circle were identified, the percentage of disruption at the same site was calculated according to the counts among the total chimeric reads.

### Verification of CvBV host integration motifs

3 CvBV circles (CvBV_02, 12 and 22) were randomly chosen to confirm host integration motifs (HIMs) identified by chimeric reads according to the PCR-based detection [18]. Briefly, CvBV_02, 21 and 22 were divided into 2, 7 and 8 segments, respectively, by designing overlapping primer pairs that specifically amplified regions of different CvBV circles (S5 Data). Genomic DNA from hemocytes of parasitized *P*. *xylostella* (24 h p.p) was used as templates, while genomic DNA isolated from CvBV virions as controls. The segments containing HIMs were further divide into 3 smaller segments by designing overlapping primer pairs (S5 Data). When the locations of target HIMs were narrowed down to 1 kb, we used hiTAIL-PCR [50] for isolation of DNA junctions where a given CvBV circle had integrated and joined with flanking *P*. *xylostella* chromosomal DNA. Pre-amplification reactions (20μL) of hiTAIL-PCR were prepared, each containing 2.0 μL PCR buffer, 200 μM each of dNTPs, 1.0 μM LAD1 and LAD2 primers, 0.3 μM Tail-1 primer, 0.5 U LA Taq, and 25ng genome DNA of parasitized *P*. *xylostella*. Each 25 μL primary hiTAIL-PCR contained 2.5 μL PCR buffer, 200 μM each of dNTPs, 0.3 μM AC1 and Tail-2 primer, 0.6 U LA Taq, and 1 μL 40-fold diluted pre-amplified product. Each secondary 25 μL hiTAIL-PCR contained 2.5 μL PCR buffer, 200 μM each of dNTPs, 0.3 μM AC1 and Tail-3 primer, 0.6 U LA Taq, and 1 μL 10-fold diluted primary hiTAIL-PCR product. The PCRs were performed with thermal conditions shown in S8 Table. Primers used for hiTAIL-PCR were shown in S5 Data. The amplified products were analyzed on 1.0% agarose gels, and single fragments were recovered from the gels and purified for sequencing.

### Structural and functional analysis of HIMs

Alignment analyses of HIM regions were performed with MEGA 7.0 software, and the visualizations of the alignments were made using Jalview (v2.10.4b1) [51]. Then, we constructed the smallest Conservative-Broken CvBV circle (CvBV_02) and its mutation circles *in vitro*. Briefly, CvBV_02 was divided into 2 fragments (SA and SB). We then cloned 2 fragments into pGEM-T vectors by using an In-Fusion HD Cloning Plus Kits (Clontech, Mountain View, CA, USA). The mutated positions were shown in Fig 3B and the mutations were achieved by subcloning using mutagenized primers (S5 Data). The recombinant CvBV_02 was extracted from *E*. *coli*. About 10^8^ copies of recombinant CvBV_02 were mixed with 1 μl Cellfecti II Reagent (Thermo Fihser). And 0.05 μl mixture was used for injection into middle 3^rd^ instar *P*. *xylostella* larvae. The integration index was calculated as the percentage of reduced products of amplifying HIM regions from CvBV_02, which was determined using specific primers with qPCR analysis (S5 Data) and the WIM region of CvBV_02 was used as an internal control. Each qPCR reaction was performed for at least three biological replicates.

### Detection of the time courses of CvBV integration

Genomic DNA of *P*. *xylostella* was isolated individually at 0 h, 0.5 h, 1 h, 2 h and 4 h p.p to determine the dynamic integrations of CvBV circles. The integration dynamic was determined through the integration indices of CvBV circles. As mentioned above, the integration index of CvBV circles were defined as the percentage of reduced products of amplifying HIM regions, which can be detected using two pairs of specific primers (S5 Data) across their HIM and WIM regions via qPCR analysis. For sample, the C_T (H)_ and C_T (W)_ indicate the fractional cycle number at which the PCR products of amplified HIM region and WIM region reach a fixed threshold, respectively. Integration index of each CvBV circle = (1-2^-ΔΔCT^)×100%. Here, ΔΔCT = ΔCT_different hrs_—ΔCT_0 h_ and ΔCT = C_T (H)_—C_T (W)_.

### RNAi of CvBV integrase genes

After analyzing the provirus genome of CvBV, we found 5 integrases genes, which were supposed to be involved in CvBV integration. The online programs InterPro (http://www.ebi.ac.uk/interpro/) was used to predict domains in these 5 integrases. They were *CVE02117*, *CVE05481*, *CVE07898*, *CVE05936* and *CVE06814* (S2 Data). T7 RiboMAX Express kit (Promega, Madison, WI, USA) was used for production and purification of double stranded RNA (dsRNA) according to the manufacturer’s instructions. Briefly, DNA fragments of ~500 bp in size were amplified by PCR from *CVE02117*, *CVE05481*, *CVE07898*, *CVE05936*, *CVE06814* and *GFP* gene. Forward and reverse primers contained T7 promoter sequences at their 5’ end for in vitro RNA synthesis (S5 Data). 500 ng dsRNA of *CVE02117*, *CVE05481*, *CVE07898*, *CVE05936*, *CVE06814* and mixture (*CVE05481*, *CVE05936* and *CVE06814*) was injected into 1.5-day-old female pupae and the efficiency of the interference was determined by qPCR at the emergence of *C*. *vestalis*. After emergence, five female wasps were used to parasitize *P*. *xylostella*. Genomic DNA of five host *P*. *xylostella* from each group was isolated individually to determine the integration index of CvBV circles post CvBV-encoded integrase genes were interfered. The integration of each circles was determined using specific primers with qPCR analysis (S5 Data) as mentioned above.

### Transcription mRNA sequencing and data analysis

Total RNA of hemocytes from 500 parasitized *P*. *xylostella* (4 h p.p) was isolated using Trizol method (Invitrogen, Carlsbad, CA, USA) according to the manufacturer’s instruction and that isolated from non-parasitized larvae served as controls. A total amount of 1μg qualified RNA per sample was used as input material for the library preparation. The sequencing libraries were generated using the VAHTS mRNA-seq v2 Library Prep Kit for Illumina (Vazyme, China) following manufacturer’s recommendations. The clustering of the index-coded samples was performed on a cBot Cluster Generation System (Illumia, San Diego, CA, USA) according to the manufacturer’s instructions. After cluster generation, the library preparations were sequenced on an Illumina Hiseq X Ten platform and 150bp paired-end module.

*P*. *xylostella* genome index was built using Bowtie (v2.1.0) [52], and paired-end clean reads were aligned to *P*. *xylostella* genome using TopHat (v2.1.1) [53]. S6 Table shows a summary of the read counts per library. Cuffdiff (v2.2.1) [54] was used to calculate FPKMs for coding genes in each group. Gene FPKMs were computed by summing the FPKMs of the transcripts. FPKM stands for “fragments per kilobase of exon per million fragments mapped”, and it is calculated based on the length of the fragments and the reads count mapped to each fragment.

Differential gene expression analyses were performed using the edgeR package. Genes with false discovery rate (FDR) below or equal to 0.05 and fold-change variation of at least 2× were considered differentially expressed. Genes belonging to each cluster were submitted to Gene Ontology (GO) and KEGG pathway enrichment analyses to identify over-represented biological processes.

### The transcriptional profiles of host integration-related genes

qPCR was used to analyze the transcriptional profiles of integration-related genes in the host at different time points post parasitization. RNA was isolated from *P*. *xylostella* at 0 h, 0.5 h, 1 h and 2 h p.p. First strand cDNAs were synthesized using the ReverTra Ace qPCR RT kit (Toyobo, Osaka, Japan) according to the manufacturer’s instructions. The qPCR reactions were conducted as mentioned above and the primers were listed in S5 Data.

### RNAi of host *PxIN1* and *PxIN2*

dsRNA of *PxIN1* and *PxIN2* was synthesized as mentioned. To increase dsRNA stability and facilitate dsRNA delivery, injection of dsRNA was carried out with a 1:1 volume ratio of Metafectene PRO transfection reagent (Biontex, Martinsried, Germany) after incubation for 30 min at 25°C. 500 ng dsRNA was injected into middle 3^rd^ instar larvae of *P*. *xylostella* and the efficiency of the interference was determined by qPCR at 4 h post dsRNA injection. *P*. *xylostella* larvae which were treated with *PxIN1* or *PxIN2* dsRNA, was parasitized by *C*. *vestalis*. Genomic DNA of *P*. *xylostella* was isolated individually to determine the integration indices of CvBV circles after *PxIN1* and *PxIN2* were interfered. The integration indices were determined as mentioned above.

### Recombinant baculovirus rescue experiments

The pFASTBAC-HTb (Invitrogen, San Diego, CA, USA) vector for baculovirus expression in *P*. *xylostella* hemocytes was modified by the insertion of open reading frames (ORFs) of target genes using conventional molecular biology techniques. To engineer recombinant baculovirus, cDNA encoding *GFP*, *CVE02117*, *CVE05481*, *CVE07898*, *CVE05936*, *CVE06814*, *PxIN1*, and *PxIN2* were amplified by PCR using specific primers (S5 Data). Recombinant baculovirus was produced with the Bac-to-Bac Baculovirus Expression System (Invitrogen, San Diego, CA, USA) according to the manuscript. The proper titer of the high concentration virus stock was determined with a viral plaque assay according to the recommendations of the Bac-to-Bac Baculovirus Expression System (Invitrogen, San Diego, CA, USA).

The RNAi experiments of 3 CvBV integrases (*CVE05481*, *CVE05936*, and *CVE06814*) and 2 host integrases (*PxIN1* and *PxIN2*) were performed as mentioned above. 10^4^ pfu baculoviruses modified by the insertion of *GFP*, *CVE05481*, *CVE05936*, *CVE06814*, *PxIN1*, and *PxIN2* were injected into *P*. *xylostella* larvae 12 h before parastization. Genomic DNA of parasitized *P*. *xylostella* was isolated individually to determine the integration indices of CvBV_04, 12, and 24 after *PxIN1* were interfered. The integration indice of CvBV_21 was determined after *PxIN2* were interfered. The integration indices of CvBV_17 and CvBV_22 were determined after *CVE05481* were interfered. The integration indice of CvBV_20 was determined after *CVE05936* were interfered. The integration indice of CvBV_06 was determined after *CVE06814* were interfered. The integration indices of CvBV_15 and CvBV_24 were determined after *CVE05481*, *CVE05936* and *CVE06814* were interfered simultaneously. The integration indices were determined as mentioned above.

### Genomic structure analyses of viral and host integrases homologues

To explore the homologues of viral and host integrases genes in other insects, we generated BLAST databases using the official gene set of each of the 171 invertebrate reference species (further details and accession numbers, please refer to S6 Data) using the DIAMOND-BLAST software with e-values, k, length and identity were set to 1-e5, 100000000, 500 AA and 40% respectively. In addition, the online programs InterPro (http://www.ebi.ac.uk/interpro/) was used to check the predicted domains in these putative homologous proteins. The proteins which showed the presence of the same domains’ arrangement were identified as homologues.

### Detection of CvBV infection in *C*. *vestalis* non-adaptive hosts

We also detected the infections of CvBV in non-adaptive hosts of *C*. *vestalis*, with or without homologous of *PxIN1* and *PxIN2*. For caterpillar hosts, middle 2^nd^ instar larvae of *Spodoptera litura*, *Bombyx mori*, *Helicoverpa armigera* and *Chilo suppressalis* were injected with 0.05 female equivalent (FE) CvBV, which is a dosage close to real parasitism [55]. Genomic DNA of each caterpillar hemocytes were isolated at 24 h p.i to determine the integration index of CvBV_04, 12, 21 and 24. The four Lepidoptera species were all maintained at Institute of Insect Sciences, Zhejiang University.

While for *Drosophila melanogaster*, the stocks were raised on standard cornmeal/yeast/agar medium at 18°C. *W*^*1118*^ was used as the wild type stock and the Bloomington stocks *Hml-GAL4* (BS#8700) was also used in this study. To obtain the PxIN1 and PxIN2 transgenic lines, the *PxIN1* and *PxIN2* gene was firstly cloned into the pUAST-attb vector [56]. The transgenic *Drosophila* line carrying the *UAS-PxIN1* or *UAS-PxIN2* gene was obtained by phiC31 integrase-mediated insertion into the attP2 landing-site locus on the third chromosome. For the CvBV infection experiments, hemocytes specific expression line *Hml-GAL4* were crossed at 25°C with *UAS-PxIN1* or *UAS-PxIN2* to drive ectopic expression of PxIN1 or PxIN2 in hemocytes, respectively. *Hml-GAL4* were crossed with *W*^*1118*^ as control. Middle 2^nd^ instar *Drosophila* larvae were injected with 0.01 FE CvBV. Flies were kept at 25°C for 24 h. Genomic DNA of fly hemocytes was isolated 24 h p.i to determine the integration indices of CvBV_04, 12 and 21.

### Phylogenetic analysis of orthologs of integrase 1 and integrase 2

The blastp and tblastn iterative approach was used to search for putative orthologs of *integrase 1* and *integrase 2* in insecta, bacteria and retrovirus. NCBI conserved domain search online server (https://www.ncbi.nlm.nih.gov/Structure/cdd/wrpsb.cgi) was used to identified conserved domain across all identified proteins. The proteins which showed the presence of the same domains’ arrangement were used. Alignment of *integrase 1* (IN1) RT_LTR domain and *integrase 2* RT_pepA17 (IN2) domain across all identified proteins using MUSCLE v3.8.3. These two conserved domains were used to infer the ML phylogenetic tree using RAxML. The information of PxIN1 and PxIN2 orthologs in insecta, bacteria and retrovirus were shown in S7 and S8 Data.

### Statistical analysis

All statistical analyses were performed in SPSS Statistics 20.0 software (IBM). Data had a normal distribution and are presented as means ± standard deviation (SD). All the data was analyzed by the ANOVA (one-way) Tukey-test, with significance threshold of P < 0.05.

## Supporting information

S1 Fig Updated CvBV genome for proviral segments. (TIF)

S2 Fig Location of CvBV circles on *C*. *vestalis* genome and the WIMs of all CvBV segments. (TIF)

S3 Fig Proportions of different CvBV Circles. (TIF)

S4 Fig Performance of integration sites for different CvBV circles in *P*. *xylostella* genome. (TIF)

S5 Fig IE distribution over the *P*. *xylostella* genome. (TIF)

S6 Fig PCR-based detection assay to verify HIM regions. (TIF)

S7 Fig The alignment of deleted fragments during CBCs integration. (TIF)

S8 Fig Integration indices of CvBV circles. (TIF)

S9 Fig Maximum likelihood tree based upon the *vlf-1*, *integrase* (*HzNVorf144*), and *HzNVorf140* genes in other BVs and nudiviruses. (TIF)

S10 Fig Integration indices of CBCs after knocking down CvBV integrase genes. (TIF)

S11 Fig Transcriptome analysis for identification of two host integrase enzymes. (TIF)

S12 Fig Phylogenetic analysis of integrase 1 and integrase 2 genes. (TIF)

S1 Table The basic features of the CvBV resequencing genome. (XLSX)

S2 Table The information of CvBV ORFs. (XLSX)

S3 Table The location of CvBV resequencing segments on *C*. *vestalis* genome. (XLSX)

S4 Table The basic features of sequence Rawdata of hemocytes of *P*. *xylostella* at 24h p.p. (XLSX)

S5 Table Reads numbers of CvBV circles. (XLSX)

S6 Table Summary of the read counts per library. (XLSX)

S7 Table Standard curves of CvBV WIM copies. (XLSX)

S8 Table Thermal conditions for hiTAIL-PCR. (XLSX)

S1 Data Sequences of the DNA junctions. (XLSX)

S2 Data Nudivirus-like homologs in *C*. *vestalis* genome. (XLSX)

S3 Data 15 up-regulated and 7 down-regulated genes associated with DNA integration in *P*. *xylostella*. (XLSX)

S4 Data DNA Repair and Recombination Proteins(9↑ 36↓). (XLSX)

S5 Data List of primers used in this study. (XLSX)

S6 Data The genomic information of 171 invertebrate reference species. (XLSX)

S7 Data The information of PxIN1 orthologs in insecta, bacteria and retrovirus. (XLSX)

S8 Data The information of PxIN2 orthologs in insecta, bacteria and retrovirus. (XLSX)
