# Supplementary figures and images for: Bracoviruses recruit host integrases for their integration into caterpillar’s genome
Source: PLoS Genet. 2021 Sep 7;17(9):e1009751. doi: 10.1371/journal.pgen.1009751 (PMC8460044; doi:10.1371/journal.pgen.1009751)

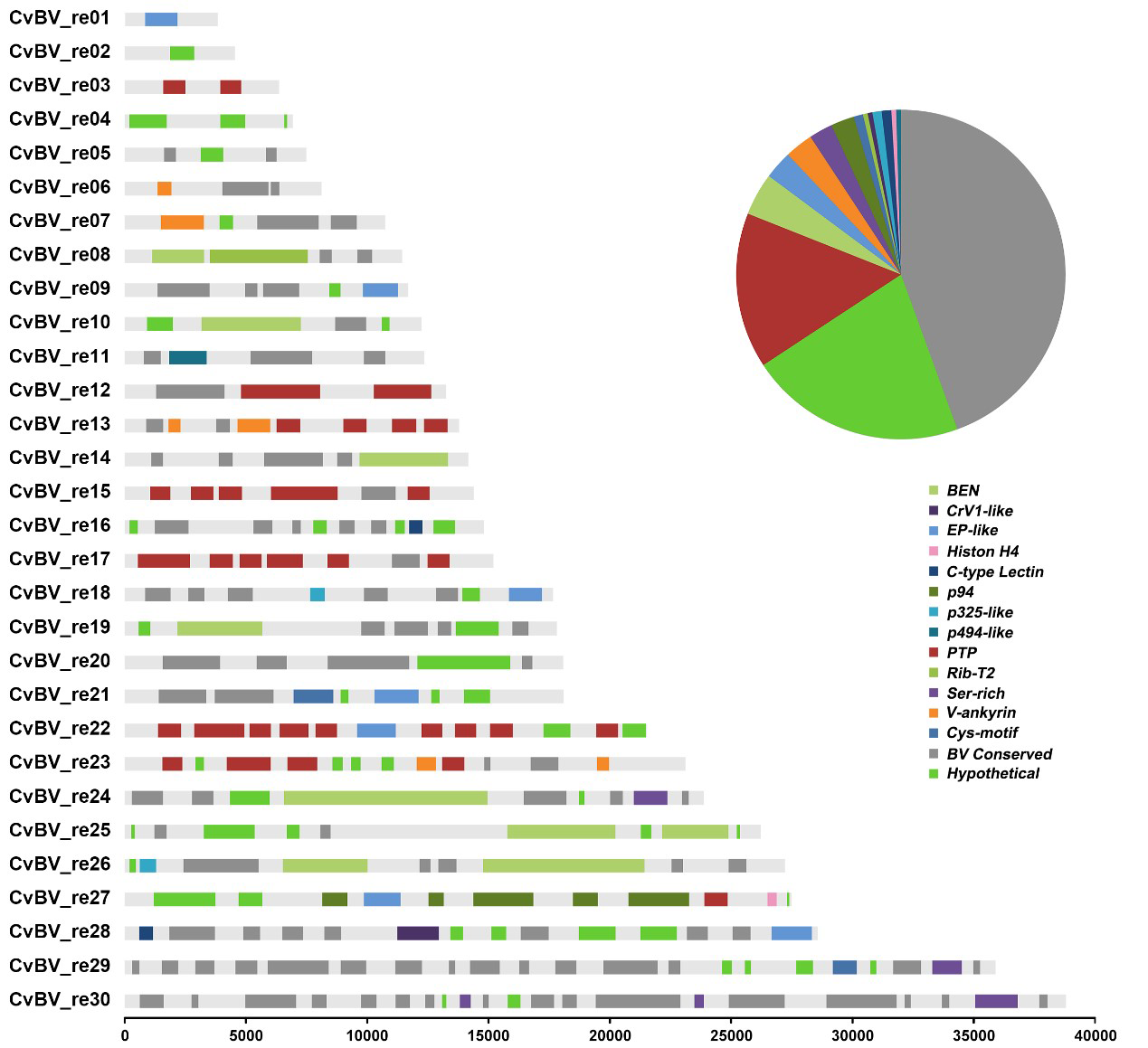

Supplement: S1 Fig — (TIF) [file pgen.1009751.s001.tif]

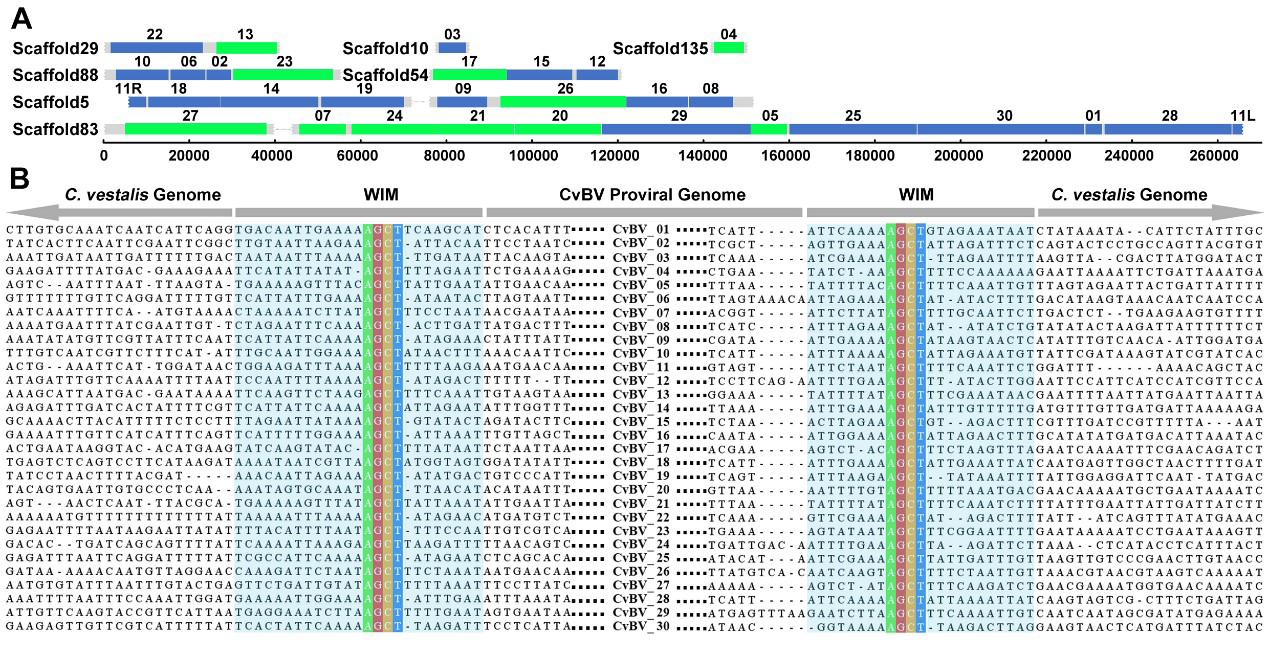

Supplement: S2 Fig — (TIF) [file pgen.1009751.s002.tif]

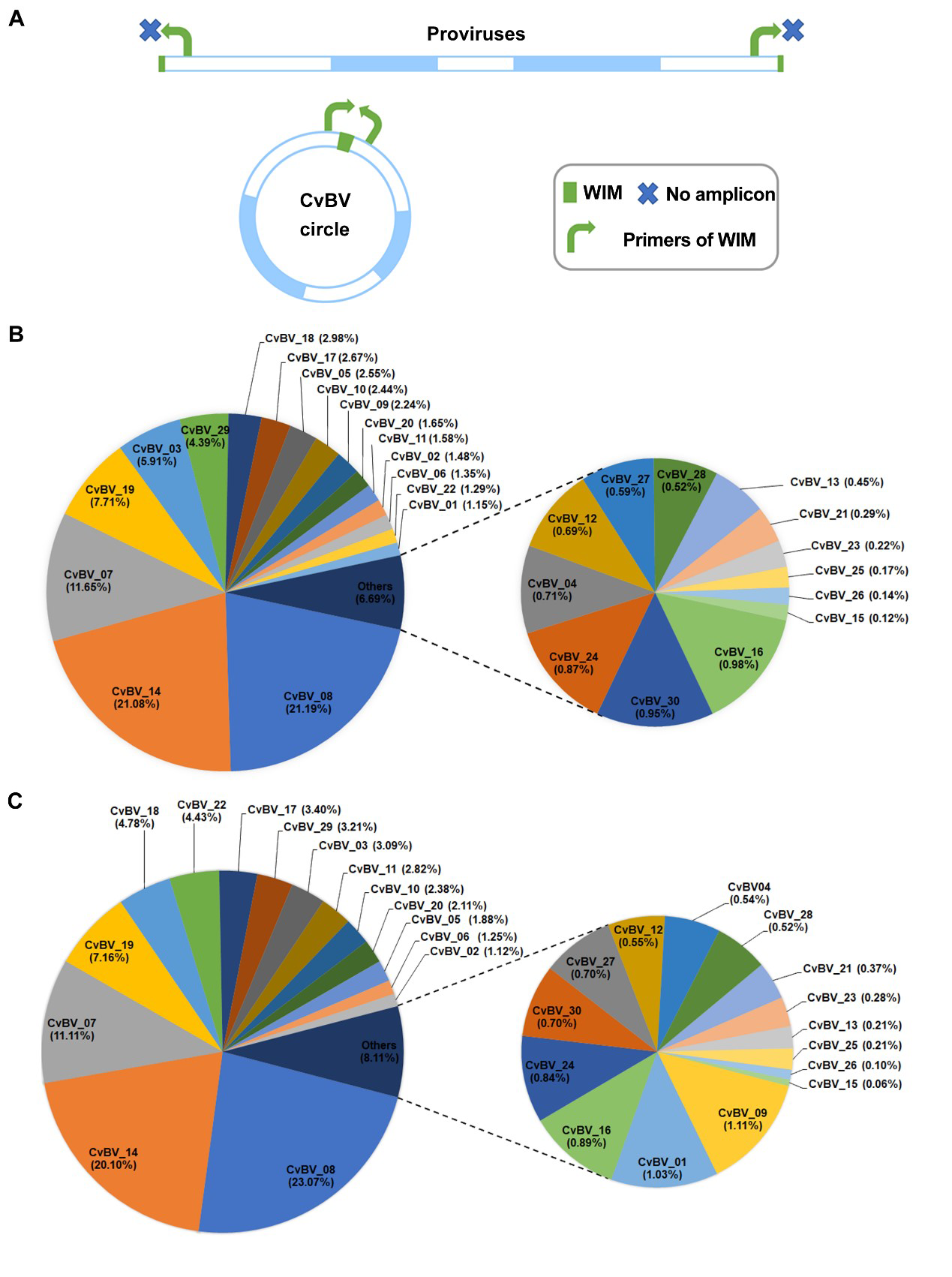

Supplement: S3 Fig — (TIF) [file pgen.1009751.s003.tif]

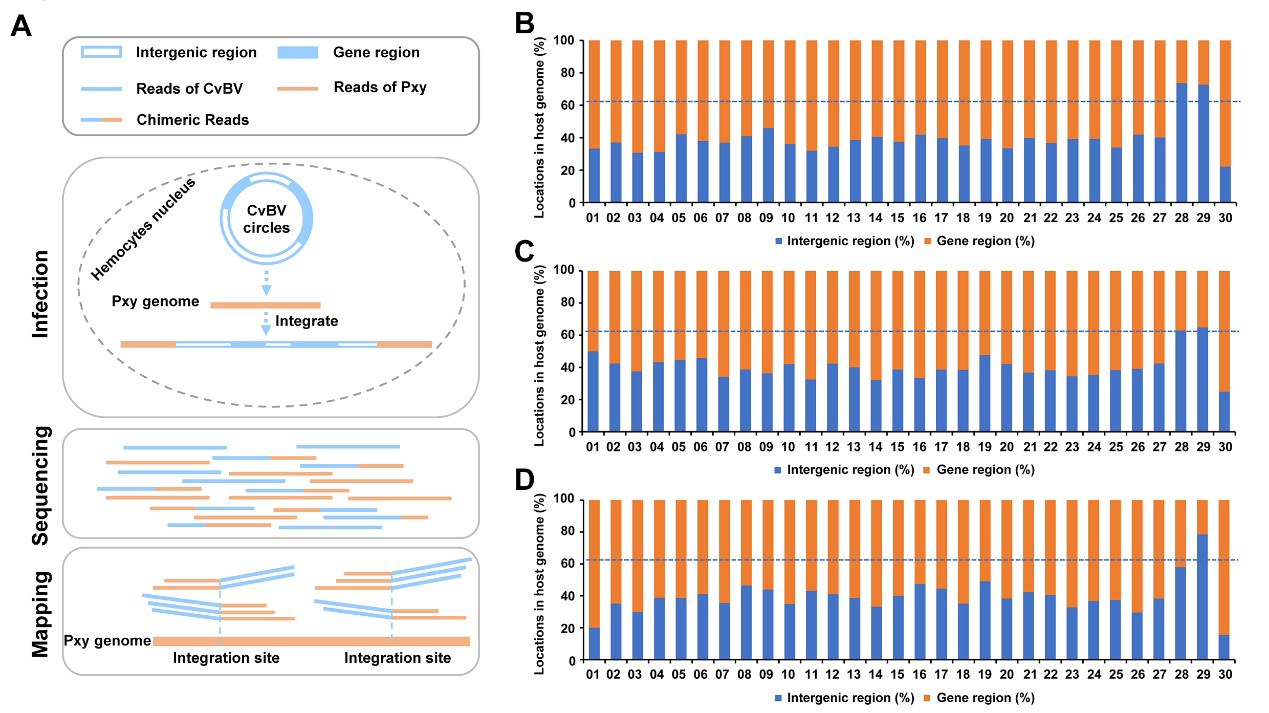

Supplement: S4 Fig — (TIF) [file pgen.1009751.s004.tif]

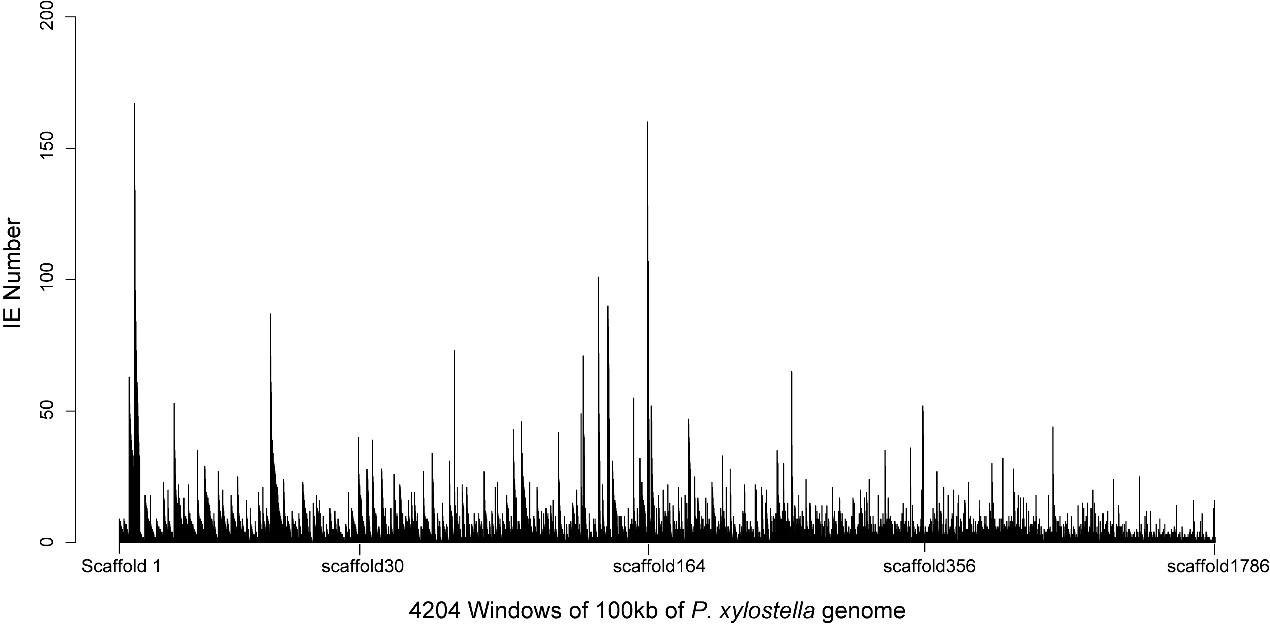

Supplement: S5 Fig — (TIF) [file pgen.1009751.s005.tif]

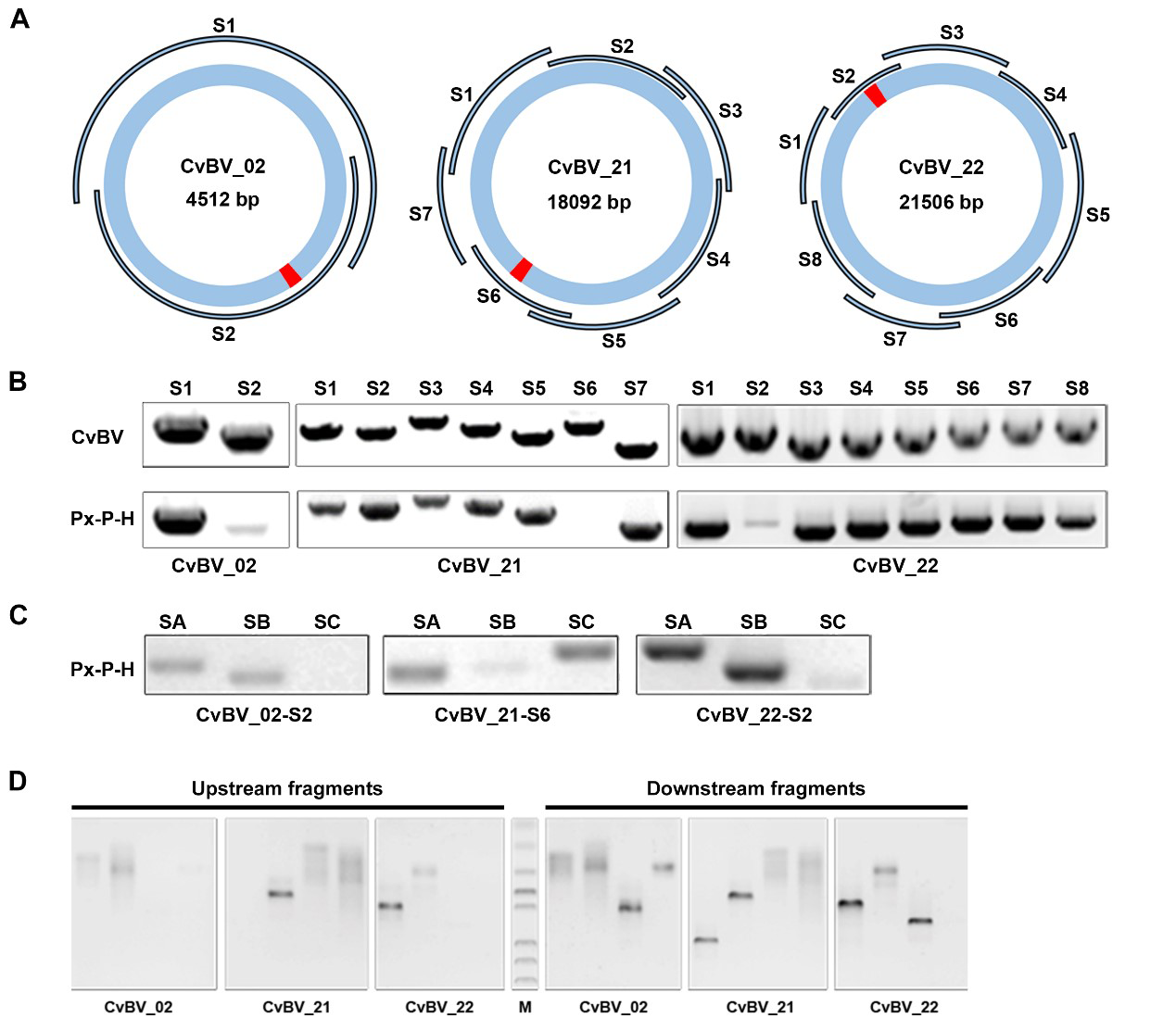

Supplement: S6 Fig — (TIF) [file pgen.1009751.s006.tif]

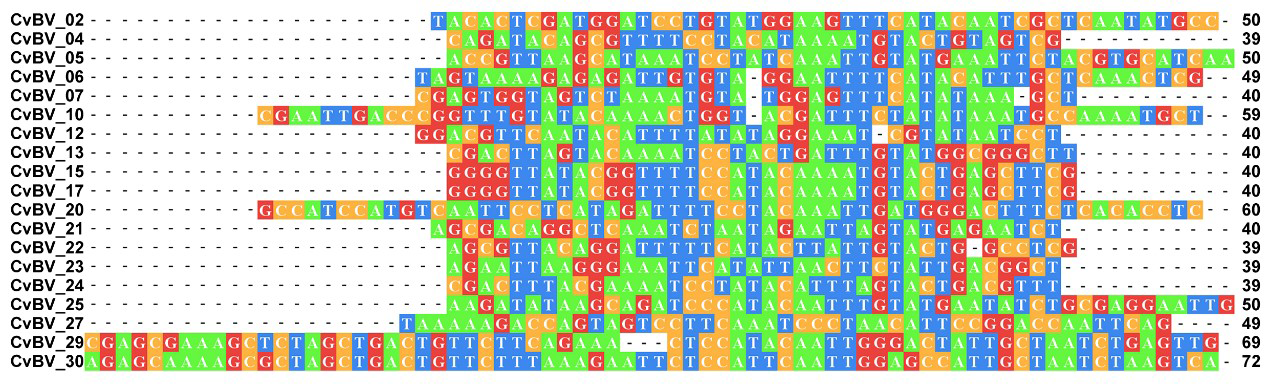

Supplement: S7 Fig — (TIF) [file pgen.1009751.s007.tif]

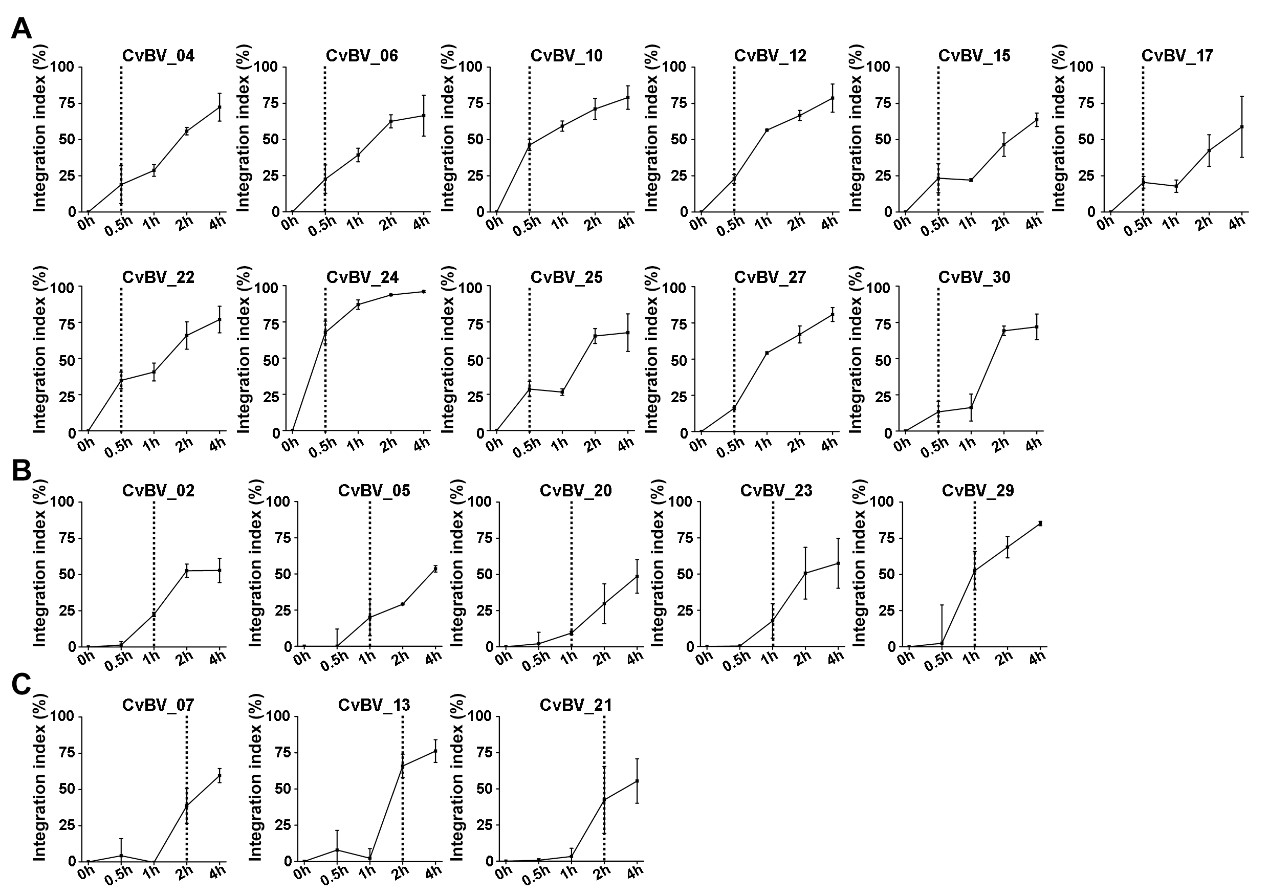

Supplement: S8 Fig — (TIF) [file pgen.1009751.s008.tif]

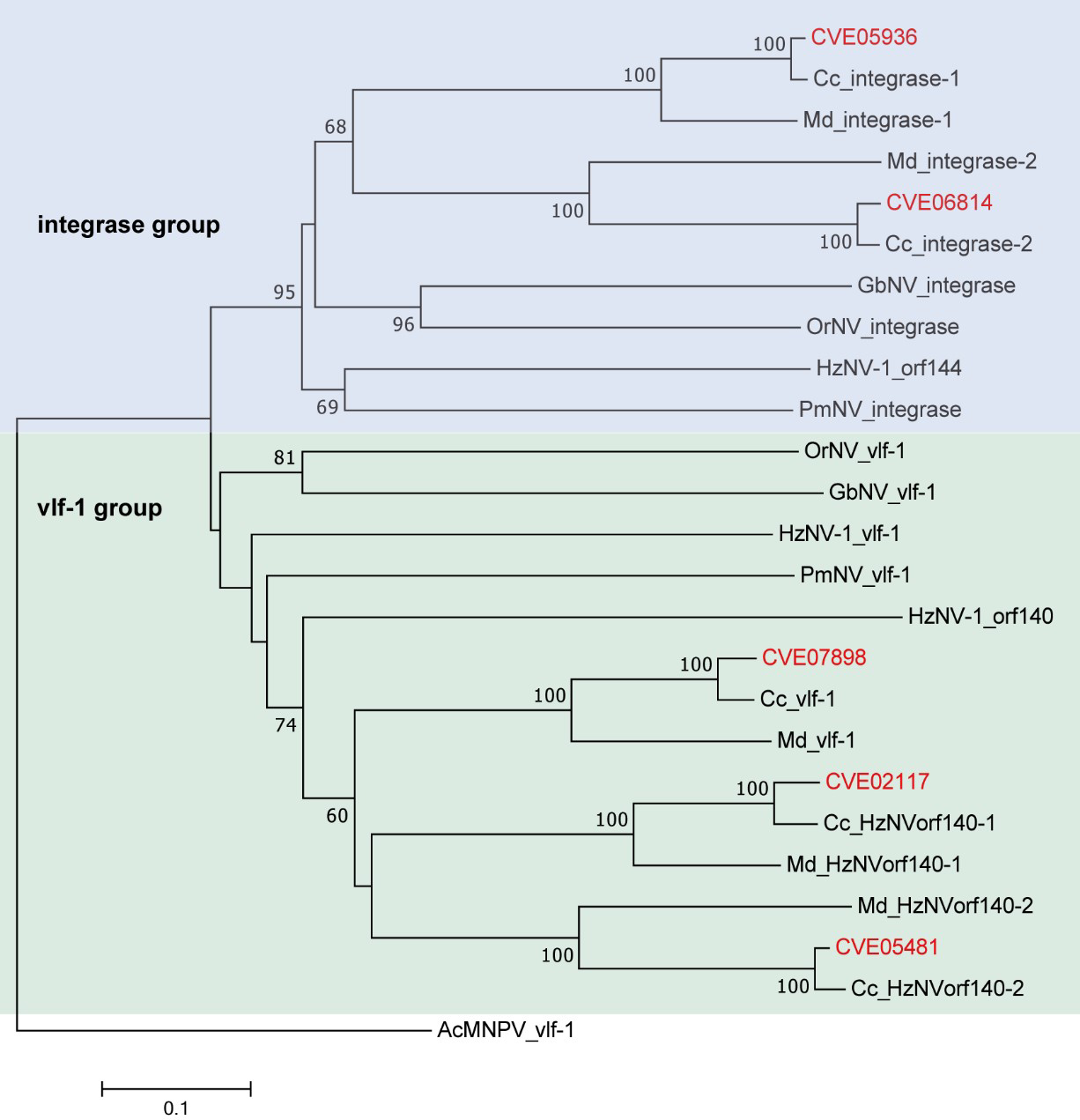

Supplement: S9 Fig — (TIF) [file pgen.1009751.s009.tif]

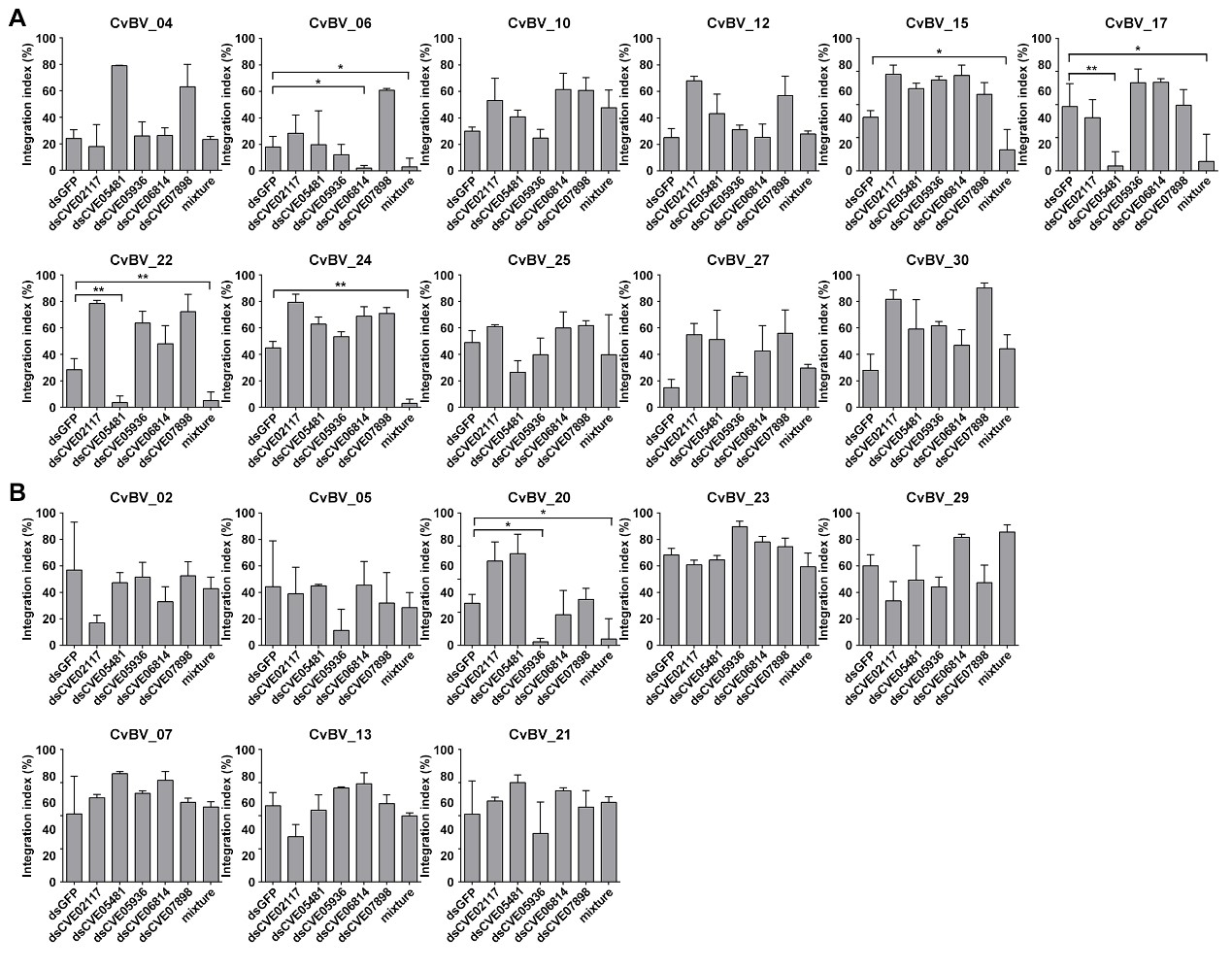

Supplement: S10 Fig — (TIF) [file pgen.1009751.s010.tif]

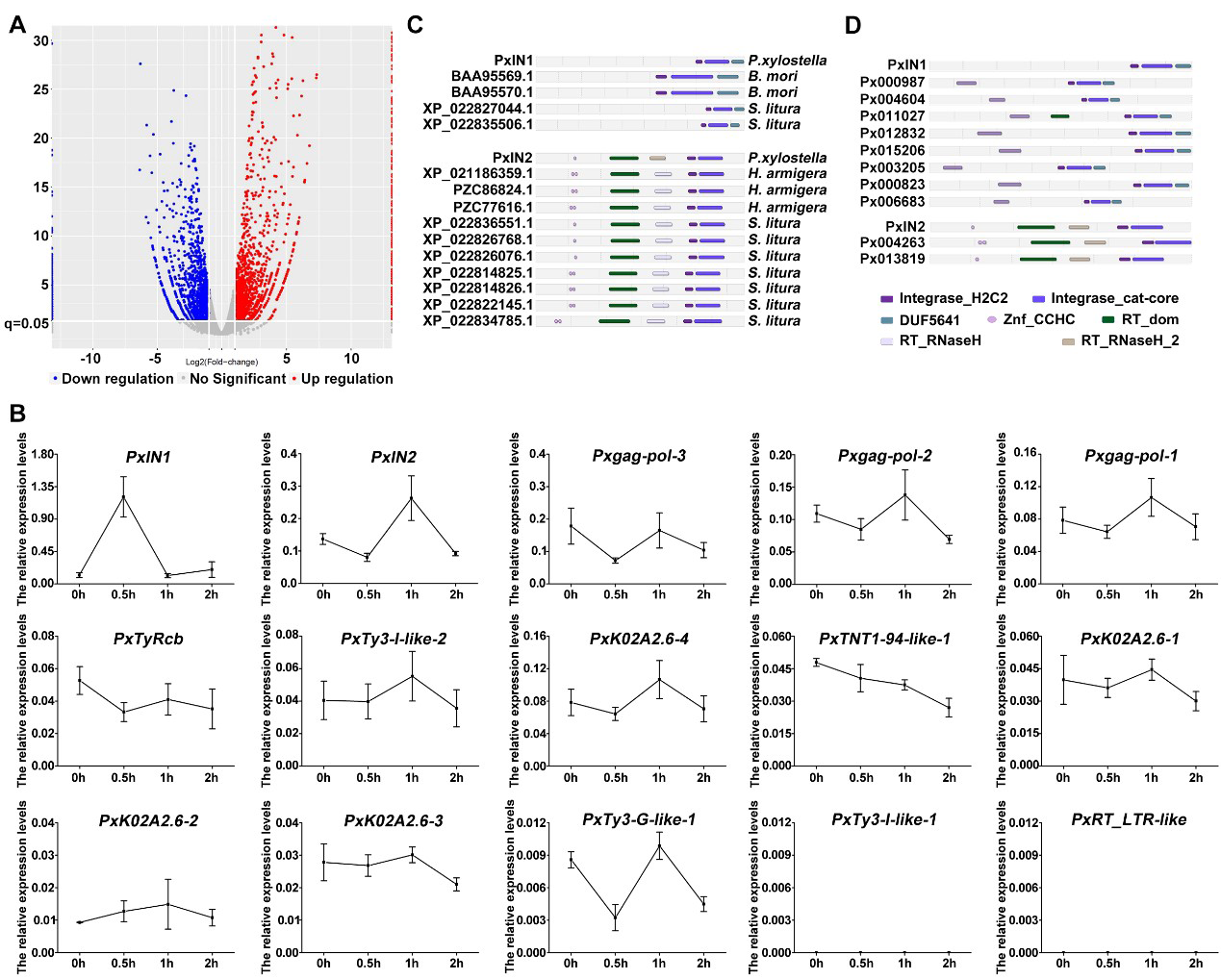

Supplement: S11 Fig — (TIF) [file pgen.1009751.s011.tif]

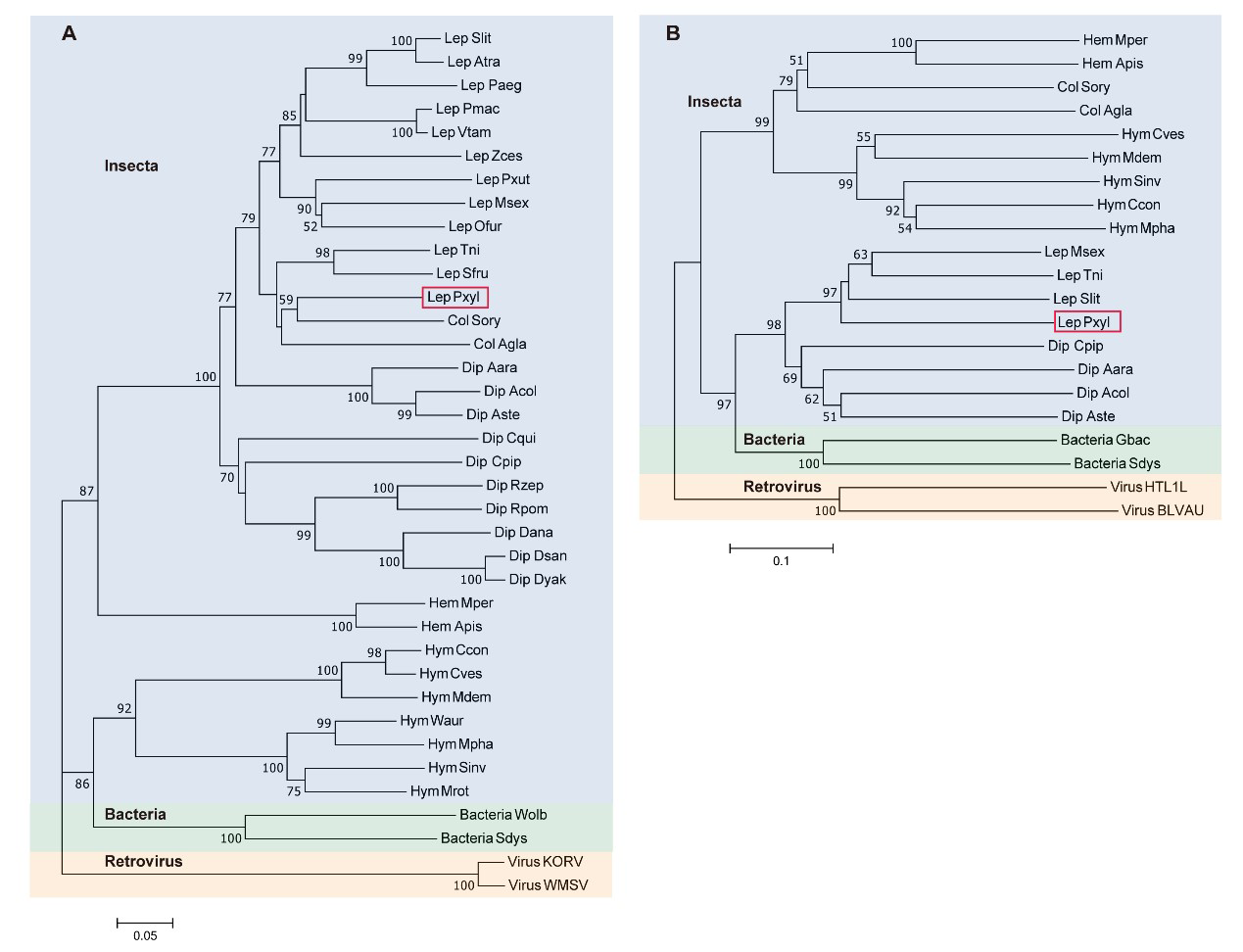

Supplement: S12 Fig — (TIF) [file pgen.1009751.s012.tif]
